# A state-space approach to understand responses of organisms, populations and communities to multiple environmental drivers

**DOI:** 10.1038/s42003-021-02585-1

**Published:** 2021-09-30

**Authors:** Luis Giménez, Adreeja Chatterjee, Gabriela Torres

**Affiliations:** 1 Biologische Anstalt Helgoland, Alfred-Wegener-Institut, Helmholtz-Zentrum für Polar- und Meeresforschung, Helgoland, Germany; 2 School of Ocean Sciences, Bangor University, Menai Bridge, United Kingdom

**Keywords:** Climate-change ecology, Ecophysiology

## Abstract

Understanding the response of biotic systems to multiple environmental drivers is one of the major concerns in ecology. The most common approach in multiple driver research includes the classification of interactive responses into categories (antagonistic, synergistic). However, there are situations where the use of classification schemes limits our understanding or cannot be applied. Here, we introduce and explore an approach that allows us to better appreciate variability in responses to multiple drivers. We then apply it to a case, comparing effects of heatwaves on performance of a cold-adapted species and a warm-adapted competitor. The heatwaves had a negative effect on the native (but not on the exotic) species and the approach highlighted that the exotic species was less responsive to multivariate environmental variation than the native species. Overall, we show how the proposed approach can enhance our understanding of variation in responses due to different driver intensities, species, genotypes, ontogeny, life-phases or among spatial scales at any level of biological organization.

## Abstract (web summary)

Giménez et al. explore a “state-space” approach (SSEA) to examine variation in effects of multiple environmental drivers on biological systems. They illustrate the SSEA with a case study where larvae of an exotic crab were less responsive to an experimental heatwave than those of a native species.

## Introduction

**Fig. 1:**
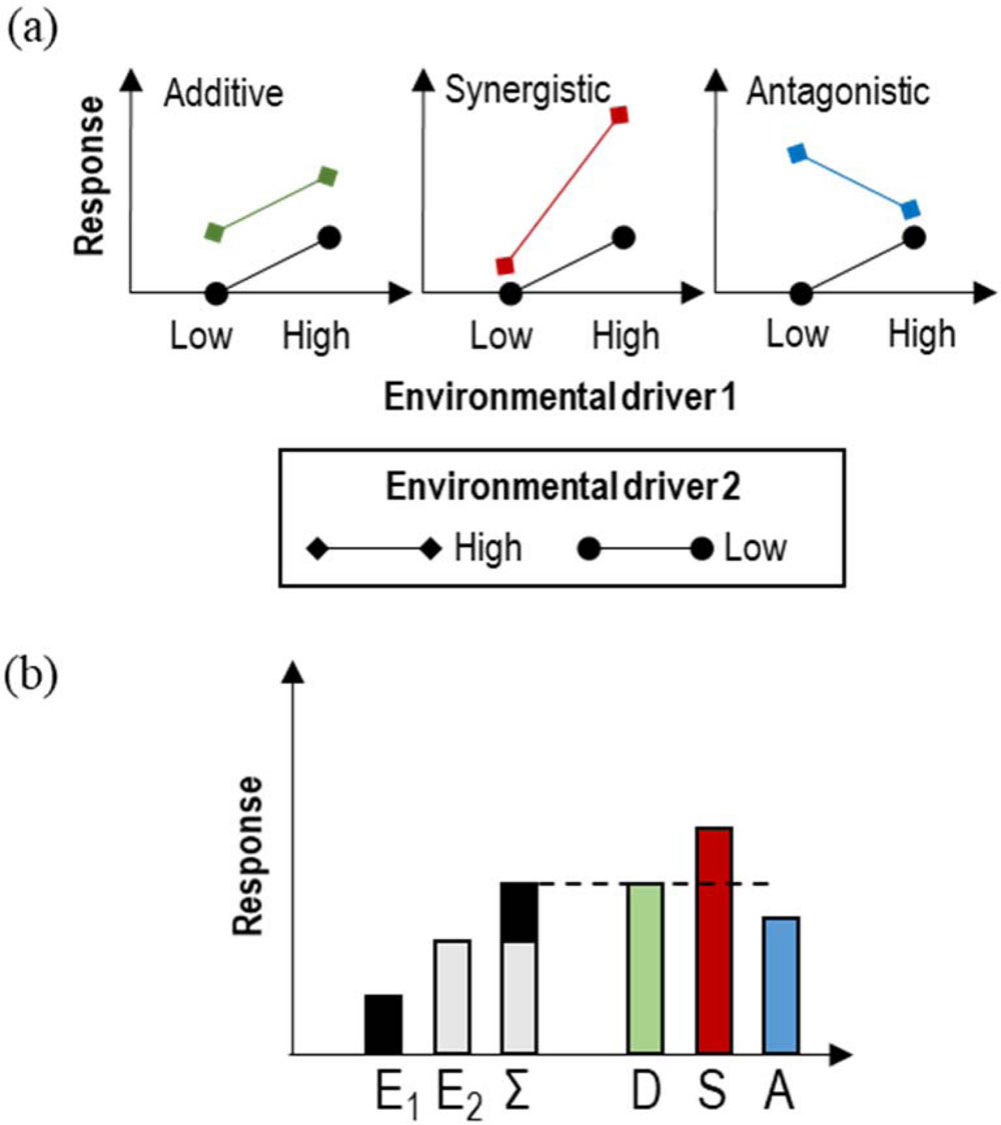
Graphical representations of interactive and additive effects for a case of double positive effects of two environmental drivers (E_1_ and E_2_) on a biotic response. **a** Interaction plots corresponding to additive, synergistic and antagonistic responses. **b** Bar charts comparing additive and interactive responses (D: Additive, S: Synergistic, A: Antagonistic), by adding the isolated response to E_1_ and E_2_, abbreviated as Σ. More types of responses are found in Crain et al.^6^ and Piggott et al.^10^.

As a consequence of anthropogenic change, biotic systems must cope with multivariate alterations in natural habitats; for instance, organisms are now being exposed to increased temperature combined with habitat loss^1^, food limitation^2^, pollutants^3^, ocean acidification^4^ and deoxygenation^5^. Over the past 20 years, a series of reviews have shown that the combined action of several environmental variables (here called ‘drivers’ but also called ‘stressors’ in older literature) cannot be predicted from the additive effects of each driver acting in isolation^6–8^. Such interactive effects are usually classified into one of many categories, which are summarised here as, additive, synergistic or antagonistic (Fig. 1). Thus, an approach to study multiple-driver responses consists of quantifying the magnitude of responses and then assign them to the above categories (‘classification of responses’: thereafter called CAR). This is important to better understand how biological systems respond to both anthropogenic influence and environmental change^6,9–11^. The identification of responses is also important to assess how management can mitigate the negative effects of climate change^12^. Synergistic effects for instance, represent situations where the action of a driver exacerbates the action of a second driver, beyond the response expected for an additive effect, e.g. effects of increased temperature under scenarios of food limitation or in case of habitat loss. Such effects are critical in accelerating the collapse of a biological system^13^; however, the mitigation effect produced by management actions on only one of the drivers (e.g. create new habitat) is predicted to be strong. Antagonistic effects represent situations where a driver mitigates or reverses the effect of a second driver; hence the response is smaller than expected from an additive effect. Antagonism occurs for instance when organisms are physiologically adapted to experience the drivers in combination (cross-tolerance:^14^), or in communities where species’ tolerances are positively correlated (positive co-tolerance:^15^). Multiple-driver research is essential for understanding responses to climate change and other environmental shifts, but it is currently facing several challenges. For instance, there are logistical difficulties in assessing and understanding responses to multiple environmental drivers^16^, and there is a need to offer mechanistic explanations for the development of predictive models^17^.

Another challenge in multiple-drivers research concerns evaluation of the generality of responses to climate change^18,19^, i.e. the range of spatial, or temporal scale, or the biological level at which a particular response occurs^20^. The evaluation of generality in the response of a biological system demands the repetition of experiments over appropriate scales and biological units^18,19,21^. For example, at the species level, current models are beginning to integrate functional traits^22,23^ to determine which species will reshape patterns of biodiversity and ecosystem services. However, for a given species, responses to climate drivers may vary among and within populations^24–28^ reflecting both genetic and environmental variation^25,29^. Within and among-population variation occurs at different spatial scales and represents an important source of variation in population growth, community dynamics and responses to climate change^22,30^. The same idea applies to the community level, where the magnitude of disturbance and species interactions vary at several spatial scales^31–34^. The common problem at all those ecological levels is that the average response over experimental repetitions (e.g. across spatial scales or local populations) may not tell the full story; by focusing on interpreting results from only the overall mean response we may miss important pieces of information. In those situations, one may gain more insight in appreciating the variation in the responses^25,30,31,35^.

Here we address the challenge of appreciating variation in multiple-drivers research by proposing and exploring a ‘state-space’ approach (for simplicity, abbreviated as ‘sSEA’) to quantify and compare the magnitude of responses to combinations of environmental drivers across experimental units. We first present the SSEA and apply it to two examples along with CAR. Second, we apply the SSEA to a case study where CAR cannot be applied. This case study compares the effects of an experimental heatwave and other environmental drivers on the performance of offspring of a cold-adapted crab and a warm-adapted competitor, where we cannot define a single control treatment for both species. Overall we show that one can apply SSEA and classify responses into categories and that SSEA can be applied for situations where CAR is not suitable. In the following sections, we present the state-space approach and apply it to our study system.

## Results

### A state-space approach (SSEA) to quantify responses to multiple-drivers

For simplicity, here we consider a case with two quantitative drivers, *E*_*1*_*, E*_*2*_, (e.g. temperature and food level). The effects of the drivers are analysed through an orthogonal-factorial experiment quantifying the average response to driver combinations *R*(*E*_*1*_*, E*_*2*_). Hence, our approach is valid for cases where factorial experiments are feasible and might be extended to more than two factors. However, this is not always the case because higher-order factorial experiments become intractable or unfeasible^16^. Thus, the SSEA is not meant to substitute approaches to multiple-drivers research where factorial experiments are not logistically possible, but instead as an additional tool for cases where factorial experiments are applicable.

We also assume that the response is quantified in the appropriate scale: for instance, in case of the proportion of survival, the data are analysed in the logarithmic scale, which transforms a multiplicative model to an additive model^7^; the multiplicative model is a type of ‘null model’ used in multiple-driver research as a way to infer the joint effect of such drivers^36^; some have been used to infer mechanisms although recent analysis suggest that, at least for the levels of community and ecosystem, it is ‘not possible to make strong mechanistic inferences from null models’^17^. In any case, the SSEA does not make assumptions about mechanisms.

The calculations are based on two levels of each driver, organised in four treatment combinations (2 × 2 design). The method can be applied to factorial experiments of any number of levels by forming groups of 2 × 2 designs. Each 2 × 2 design contains the average responses of control (or more generally, a ‘reference treatment’; the same for all groups: see below), defined as *R*(*0,0*), two treatment levels, quantifying single driver responses, *R*(*a,0*) and *R*(*0,b*), and a fourth treatment quantifying the ‘combined response’, i.e. the average response of the treatment where both drivers are present in their respective levels *R*(*a,b*). The additive effect is calculated as the sum of each separated effect; defining the differences *D*_*1*_(*a*) *=* *R*(*a,0*) − *R*(*0,0*) and *D*_*2*_(*b*) *=* *R*(*0,b*) − *R*(*0,0*) as the magnitude of the separate effects, the additive effect is given by *A*(*a,b*) *=* *D*_*1*_(*a*) *+* *D*_*2*_(*b*). In addition, deviations from the additive effect are quantified as *G*(*a,b*) *=* *R*(*a,b*)*–A*(*a,b*) − *R*(*0,0*).

**Fig. 2:**
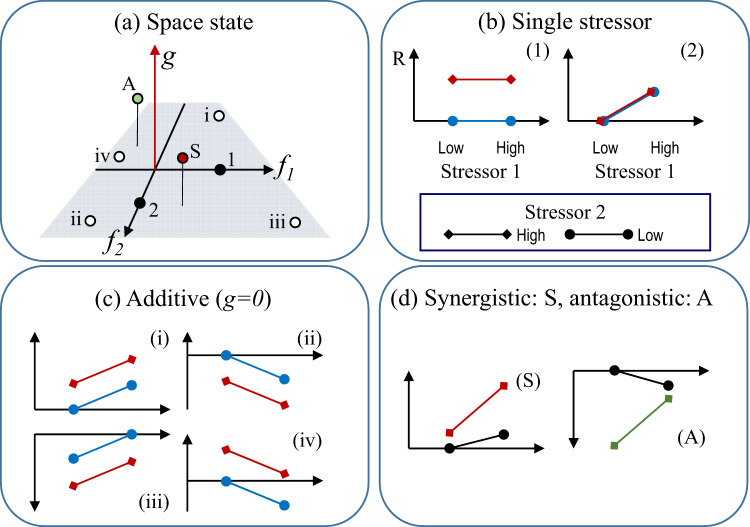
The state-space representation and the mapping of selected types of multiple-driver responses into the state-space. **a** The state-space representation is based on plotting the response *R*(*a,b*) as its three components: isolated effect of driver 1: *D*_*1*_(*a*) *=* *f*_*1*_; isolated effect of driver 2: *D*_*2*_(*b*) *=* *f*_*2*_; combined driver effect: *G*(*a,b*) *=* *g*. Single driver responses shown in (**b**) are mapped in (**a**) on the axes *f*_*1*_ and *f*_*2*_, respectively. Points corresponding to additive responses (**c**) lie on the plane defined by *f*_*1*_ and *f*_*2*_ marked in grey; they are defined as double positive (i), double negative (ii) and responses of different directions (iii and iv). **d** Selected interactive responses: synergistic for the double positive effect and antagonistic for the double negative. Other interactive responses (not shown) would be projected downwards (negative *g* value) or in the quadrants defined by responses of different signs (either *f*_*1*_ or *f*_*2*_ negative).

One of the methods used to classify responses is to compare *G*(*a,b*) vs *A*(*a,b*)*:* in geometrical terms, this is equivalent to representing the responses into a one-dimensional space with a single axis where the origin is given by the additive response. We propose a representation in a 3D space defined by ‘state variables’ (*f*_*1*_, *f*_*2*_ and *g* in Fig. 2a). There is the possibility to consider responses to any number of drivers (in a high dimensional representation), but such cases are not studied here. The coordinates in the space are given by the contribution, *D*_*1*_(*a*)*, D*_*2*_(*b*) and *G*(*a,b*), i.e. of each separate driver to the response, plus the contribution of the joint effect; hence, for *a,b* as driver levels we have *f*_*1*_(a) = *D*_*1*_(*a*)*, f*_*2*_(*b*) = *D*_*2*_(*b*)*, g*(*a,b*) *=* *G*(*a,b*). If a study compares *n*-populations of organisms of a given species, a factorial experiment is carried out on each population, and the output of the experiment is represented as a collection of *n*-points in such space. The above-defined state variables correspond to values of polynomials with an independent contribution to the response of two drivers combined (Supplementary Note 1 and Supplementary Fig. 1). Because polynomials represent linearly independent vector space, *f*_*1*_*, f*_*2*_ and *g* constitute linear basis expansion in *E*_*1*_ and *E*_*2*_ (see ref. ^37^ pp. 139–140). Therefore, *f*_*1*_*, f*_*2*_ and *g* define a linear space and any response, *R*(*a,b*), can be represented graphically on it (Fig. 2a). Because in such ‘space’, we can plot the ‘state’ of the system after it is exposed to two different drivers, we refer to it as a ‘state-space approach’. This name matches the definition of state-space^38^ as a set of conceivable values of a system. The only source of variation not captured in that space is that related to the control; however, the variation associated to the control may be considered as a fourth axis in the representation which then can be plotted as a series of 2D representations.

The SSEA representation maps the different categorical types of responses (e.g. synergistic, antagonistic as defined in Crain et al.^6^, Pigott et al.^10^) into a continuum (Fig. 2). The representation emphasises the magnitude of the responses among the units being compared, irrespective of the qualitative type of response. Thus, graphically, the SSEA resembles (although it is not!) the output of a principal component analysis (PCA), which also spans a linear space.

**Fig. 3:**
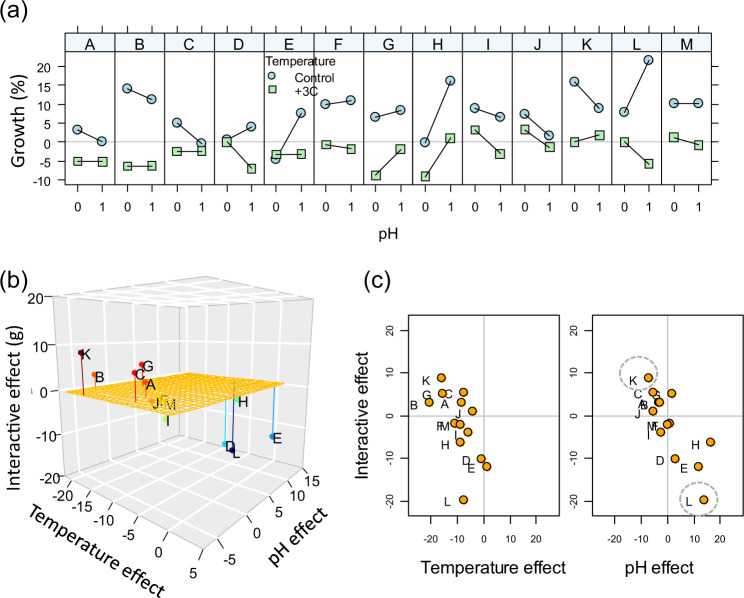
Combined effects of temperature and pH on average growth of 13 genotypes of a marine bryozoan (source: Durrant et al.^39^, Fig. 2). **a** Interaction plots of means by colony (colonies: A–M): pH levels are: 0: pH = 8.1 (control); 1: pH = 7.8. State-space representations are given as (**b**) 3D plot and (**c**) 2D projections for the planes defined by the interactive effect, and that of pH and temperature separately; there is a third projection for the plane defined by the effect of pH and temperature that is not shown here. Each point in (**b**, **c**) is calculated from the four averages represented in (**a**). The negative effect of increased temperature on growth observed in (**a**) and found by Durrant et al.^39^, is seen in (**b**, **c**) as most colonies being towards the negative side of the temperature axis. The variability in responses among genotypes (interaction genotype:pH:temperature) is well appreciated (e.g. compare colony L with K) as variations in (**1**) whether the effect of reduced pH varies between negative and positive; hence, two drivers can operate as a double negative (K) or in different directions. (**2**) Whether the response is synergistic (K) or antagonistic (L). Data, details on methods and a representation of the full data set of Durrant et al.^39^ Fig. 2. is found in the supplement.

We apply the SSEA to a case^39^ quantifying genotypic variation in the growth of a marine bryozoan under warming and ocean acidification (Fig. 3). This example (details in Supplementary Note 2 and Supplementary algorithm 1) shows the simplicity in visualising patterns and variation through the state-space representation as compared to the interaction plots, especially with many units to compare. For instance, one can appreciate that genotypic variation is so large that the action of the two drivers vary from antagonistic to synergistic, from double negative (both drivers depress performance) to operating in opposite directions. The variability uncovered by Durrant et al.^39^ is lost when the focus is given to the average responses by species. For the same study, the type of response varies with driver intensity and how that varies depends on the genotype (Supplementary Fig. 2). In Fig. 2, the representation suggests a simple pattern where the magnitude of the interactive effects correlates with the magnitude of the single driver effects. In addition, because the magnitude of multiple-driver responses is quantified, one could explore if the observed pattern correlates with e.g. underpinning physiological traits in order to further investigate the causes of such responses. In summary, along with an interaction plot, the SSEA representation may also direct further research into the causes of the observed pattern.

**Fig. 4:**
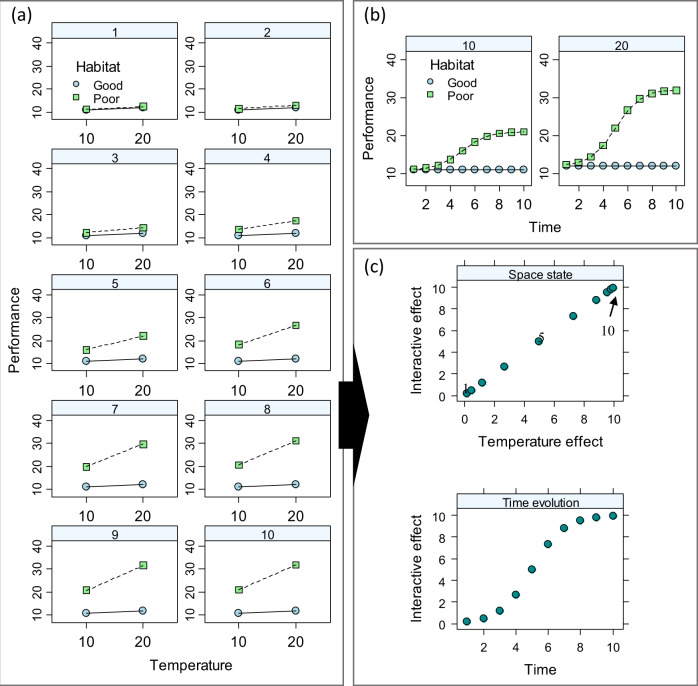
Simulation of the time evolution (in arbitrary units) of a synergistic response to temperature (°C) and a second driver indicating habitat quality (e.g. food level). The control (=best) condition is set by T = 10 °C and ‘good habitat’. In the ‘good habitat’, the increased temperature has a small effect on the response that remains constant through time. In the ‘poor habitat’, the increased temperature has a large effect that in turn increases through time following a sigmoidal pattern. **a** Interaction plot for each of the 10-time units. **b** The same data plotted over time, with separate panels for each temperature level: notice how the response in the ‘good habitat’ changes through time following the sigmoidal pattern. **c** State-space representation: upper panel: 2D plot of the interactive effect and the effect of temperature alone for each time point (time points 1, 5 and 10 are indicated); in the example, the effect of habitat quality is constant and not plotted for simplicity. In **c** notice the following: (**1**) The interactive and temperature effects are positive reflecting the synergistic pattern shown in (**a**) and the fact that both increase the response. (**2**) Low values of the interacting effect at times 1–3 reflect the small magnitude of the synergistic effect; the magnitude increases to larger values at times 4–6. Simulation details are given in Supplement, section 2.

Likewise, one may be interested in comparing responses to multiple-drivers, as the exposure time increases or in situations when organisms (or e.g. ecosystems) are exposed to drivers at different times of development (or e.g. seasons). We illustrate in a synthetic example (Fig. 4 and Supplementary algorithm 2), how the SSEA representation enables better visualisation of the time evolution of a multiple-driver response and offers the possibility to explore mechanisms driving the changes. For instance, the apparent phase shifts may coincide with life history or developmental shift (if the model system is an organism) or a shift in species composition (if the system is a community). Again here, the SSEA offers a starting point for further research and a deeper understanding of the causes of multiple-driver responses.

We must emphasise that SSEA is not the end point of the analysis; by analogy, the graphical output of a PCA is the starting point to explore the role of environmental factors in driving patterns in e.g. a local community of organisms. Another important point is that SSEA complements but does not substitute approaches based on the classification of responses or the use of standard representations based on e.g. interaction plots; instead, it is meant to be an additional tool. For correct interpretation, one must check if the observed patterns are artefacts; one can then further explore the reasons for that pattern, as discussed in the previous examples. Like in the case of the arch effect of the PCA, the patterns in SSEA may reflect artefacts. Artefacts may appear because the response variable is bounded (e.g. within 0 and 1); in such case, a re-examination of the responses in a different scale (log or logistic) may be useful. This is not an issue of SSEA alone; the issue of scale has been discussed intensively elsewhere^7,10,11^. In addition, the representation, based on *f*_*1*_, *f*_*2*_ and *g*, takes the control as a reference and in consequence, it does not explicitly highlight patterns associated to variations in the control. For example, consider a case where, across replicated experiments, the value of *R*(*0,0*) varies considerably while those of *R*(*a,0*), *R*(*0,b*) and *R*(*a,b*) remain constant; in such case, the variation of the control will be reflected in both *f*_*1*_, *f*_*2*_ and *g*. Within SSEA one can consider the control as a fourth axis of the representation and plot the response at the control vs. that quantified by *f*_*1*_, *f*_*2*_ or *g*. Notice that the variation at the control condition is biologically relevant. There might be spurious correlations *g* vs. *f*_*1*_ or *f*_*2*_ in the case of additive effects (Supplementary Note 3), associated to problems of parameter estimation; those biases are reflected in the treatment means. We propose two ways to check for artefacts (worked example in Supplement Section 3, Supplementary Figs. 3–5 and Supplementary algorithm 3). (1) Check the significance of the interaction effects or whether an interaction effect is retained in a model to decide if an observed pattern is an artefact. (2) Recode the factor codes as ‘zero-sum’ and recalculate means by fitting a linear statistical model.

### Species and context-dependent heatwaves effects

We evaluated the effects of a simulated heatwave on the performance of larvae of a cold-adapted crab *Carcinus maenas* and a warm-adapted competitor (crab *Hemigrapsus sanguineus*) co-occurring in the island of Helgoland (North Sea). These species co-occur and compete in the shores of the North Sea and North Atlantic coast of America^40,41^. For the local populations of the species, warm-adapted crabs may be able to better cope with heatwaves than cold-adapted ones; such heatwaves have become frequent worldwide^42–44^ and are producing important effects on biological systems^45–47^.

We focus on the performance of larval stages due to their vulnerability to climate-driven environmental drivers and relevance for population persistence^48^. For the case of the competing crabs, interspecific differences in larval survival may either accelerate species replacement (the warm-adapted crab is currently outcompeting the cold-adapted one^40,41^) or maintain species coexistence through a mechanism known as competition–colonisation trade-off^49^. Larvae of both species were exposed to an experimental heatwave (a steep increase in temperature: Supplementary Fig. 6) simulating conditions occurring in spring–summer when temperature increases. There were four treatments: a constant high temperature; constant low temperature; a gradual increase from low to high temperature; a sharp increase from low to high temperature. Because spring heatwaves are events of high average temperature and extreme temperature increase, their effects should be different from those caused by constant high temperature and from events of gentle temperature increase. Thus, in a factorial experiment (Supplementary Note 4 and Supplementary Fig. 6) we compared responses observed under the heatwave treatment to responses under (1) gentle temperature increase and (2) constant temperature (i.e. averages experienced in the heatwave scenario and the one experienced under gentle temperature increase). Larvae assigned to each temperature treatment were subdivided into three groups to test the performance under food limitation, low salinity and optimal food/salinity conditions. All experiments were repeated three times per species, each experiment was performed with larvae obtained from a different female.

An important point is the lack of a common optimal condition for both species. Since those species differ in the thermal optima, each one experiences the optimal temperature of the other as a ‘stressor’ (i.e. as a condition leading to a stress response). There is therefore a problem in assigning a treatment combination as a ‘control’, understood as the set of conditions not leading to stress responses (e.g. optimal temperature and optimal food condition). Because, the concept of control condition is essential to CAR, we cannot use it to compare responses among those species. Note that the categorisation of responses is not ‘invariant’ with a change of the reference treatment: the concept of ‘control treatment’ (as defined above) is essential to assign a response objectively to a specific category. If by contrast, the identification of a treatment combination as control were to be done without a consistent definition, then any study comparing or summarising types of responses would result in conclusions that are contingent on an arbitrary decision. However, one can identify a common treatment combination as ‘reference’ (= low – constant temperature) and abandon the classification system. Hence, the SSEA can be used to compare the responses with respect to a ‘reference treatment’ (i.e. not tied to a classification scheme).

**Fig. 5:**
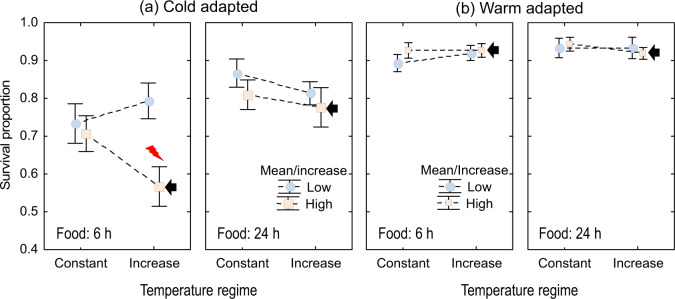
Interaction plots of the effect of simulated heatwaves on larval survival. Survival was quantified in (**a**) the native cold-adapted (*Carcinus maenas*) and (**b**) warm-adapted crab (*Hemigrapsus sanguineus*) reared in seawater under food limitation or ad libitum food conditions. The black arrow indicates the “heatwave treatment” and the red thunder in (**a**) indicates significant difference between the simulated heatwave (high mean and high rate of temperature increase) and the remaining treatments. Means are the overall mean (data from 3 females pooled) and error bars are standard errors (*n* = 15). The full set of plots of responses discriminated by female of origin and including low salinity treatments are shown in Supplementary Figs. 7 and 8.

For the cold-adapted crab, we found evidence of heatwave effect, consisting in reduced survival when larvae were exposed to conditions of limited access to food (Fig. 5a, Supplementary Note 5 and Supplementary Table 1); best models retained the interaction terms defining the heatwave effect (‘mean temperature’ by ‘temperature regime’) under food limitation (Supplementary Table 2). However, no such response was evident in the warm-adapted crab (Fig. 5b), where larval survival was consistently high (>80%); best models did not retain the interaction defining the heatwave effect (Supplementary Table 3). In the cold-adapted crab, the pattern of response to the simulated heatwave under food limitation was consistent among larvae hatching from the three tested females (Supplementary Fig. 7) although there were differences in the overall survival. In the warm-adapted crab, we did not find evidence of a ‘heatwave effect’ (Supplementary Fig. 8). Under the heatwave treatment, most mortality occurred after the temperature was increased further supporting the heatwave effect. Evidence of ‘heatwave effects’ were not found under low salinity in any of the species (Supplementary Figs. 7,8 and Supplementary Tables 4, 5).

**Fig. 6:**
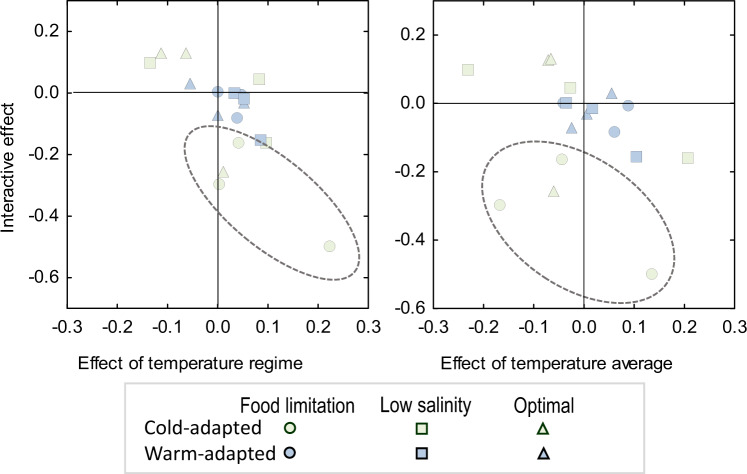
Space-state representation of the survival responses. Survival (log-transformed data) was quantified in larvae exposed to the ‘heatwave treatment’ for the native (light green symbols) and invasive crab (blue symbols). The 3D representation is decomposed in 2D plots: x-axes represent *f*_*1*_ and *f*_*2*_, i.e. giving the contribution of the temperature regime (left panel) or the temperature level (right panel); y-axis represents *g*, giving the contribution of the interactive ‘heatwave effect’ (i.e. an event characterised by high temperature in terms of average and variability). The reference condition was the treatment of low and constant temperature. Calculations were performed separately for the three environmental conditions: food limitation (but optimal salinity), optimal food access and salinity, low salinity (but optimal food access). Each point represents an average response exhibited by a group of larvae produced by a specific female and exposed to a specific combination of food and salinity. Negative values in the interacting effect correspond to decreases in survival under the heatwave treatment as compared to other temperature treatments. Dashed circles highlight responses to the simulated heatwave for the native crab. A similar representation is obtained with the data is in the raw scale (Supplementary Fig. 9).

Interspecific differences are clear in the SSEA representation (Fig. 6 and Supplementary Fig. 9) which corresponds to the results provided by 18 interaction plots (Supplementary Figs. 7, 8). Points corresponding to the warm-adapted crab cluster towards the origin of coordinates, indicating that those larvae are less sensitive to the simulated heatwave. By contrast, the distribution of points associated to the cold-adapted crab reflects the higher sensitivity to the ‘heatwave treatment’; negative values in the axis of the interactive effect, correspond to the ‘heatwave effect’ observed in larvae from the native species reared under food limitation (Supplementary Fig. 7).

We emphasise that the primary objective of this contribution is to explore a visualisation technique, but we note that interspecific differences in the sensitivity to heatwaves were consistent with expectations^45^. It is logical that a heatwave with a thermal range covering the lower limit of tolerance for the local population of the warm-adapted crab does not impair performance. In addition, the response of the cold-adapted crab, under food limitation, is consistent with studies showing resource-dependent effects of heatwaves^50–52^. It may appear striking that a heatwave of short duration and low amplitude impaired performance of the cold-adapted species, given that the amplitude of the heatwave (15–18 °C) is well within the thermal tolerance range for larvae of the local population under study (12–24 °C^53^). However, for this population, the tolerance to high temperatures drops under food limitation^54^ and a steep increase in temperature may have resulted in concomitant increases in metabolic or oxygen demands. Hence, if the results of the experiments were to represent the physiological responses of the larvae from the local population, such type of heatwaves in combination with food limitation may tilt the outcome of the competition towards the warm-adapted crab. There are however other factors that preclude us from making any general conclusion about our study. For instance, the importance of the larval tolerance on persistence in our local habitat (the island Helgoland) may depend on population connectivity and intraspecific variation in larval tolerance, sustained by genetic variation across the latitudinal range^55^. The same genetic variation is likely to lead to different outcomes of the same experiment in different populations. Hence, we take this experiment as giving an indication of that further research is needed to determine heatwaves or heat spikes can affect larval survival, recruitment and ultimately the balance of competition between the warm and cold-adapted species.

## Discussion

The development of the SSEA arose from the necessity (1) to retain information about how responses vary among replicate units (e.g. genotypes, species), driver intensity or time, and (2) to deal with situations where responses cannot be compared on the basis of classification into additive synergistic or antagonistic. Similar issues are likely to arise in attempts to quantify variation in the interactive responses across spatial and temporal scales^16,17^.

Ambiguity in the choice of a control treatment arose mainly because of differences in thermal tolerance existing among species, at least for the local populations studied in the experiment. In addition, the high survival observed in the treatment with the gentle temperature increase suggests that such treatment may be considered for the definition of control and hence points to further ambiguity. If we define the control for the cold-adapted crab as the combination of ‘low – constant’ temperature, the heatwave effect may be classified as synergistic. We have arguments for defining the ‘low’ temperature as the optimal condition in the larval thermal tolerance^53^. However, the definition of ‘constant temperature’ as optimal is based only on the general assumption that organisms perform best under constant conditions. There is however no reason to justify why the scenario of gentle temperature increase should not be considered as the ‘control’. On the contrary, if through evolutionary history, larvae have developed in springtime, when temperature increases gradually, why should those larvae be performing better under constant ‘low’ temperature than under gradually increasing temperature? Tolerance to temperature and other drivers usually varies as the system evolves (along ontogeny^55–59^); the treatment of constant temperature may expose individuals to thermal stress (continuous low temperature) at advanced stages of development. Note that treatments quantifying the effect of gentle fluctuations in temperature are needed, in order to test the ‘heatwave effect’ (e.g. based on those characterising the climatological mean). A critical point is the need to determine a threshold above which the steepness of thermal variation becomes relevant for the performance of a biological system; finding such a threshold will be essential to predict the effects of climate variability. Steepness as a heatwave trait^44^, should be critical in the spring season when temperature increases.

Issues associated to the ‘control treatment’ are resolved in the SSEA at the price of abandoning the classification scheme; the reference (constant-low temperature for both species Fig. 6) does not imply a classification of responses into synergistic or antagonistic. Additive or interactive effects (but not synergistic or antagonistic) exist independent of the ‘reference treatment’. Our treatment choice is in line with the representation given in Fig. 5, which considers food and salinity conditions as environmental contexts over which the simulated heatwave operates on the larval physiological system and modifies survival. Other representations are possible (see Supplementary Fig. 10).

In conclusion, new approaches are needed to quantify and predict the effects of climate-driven changes in biological systems. The SSEA enables us to visualise, quantify and compare the performance of biological systems across experimental units (species, genotypes, communities) when factorial designs are feasible. Because of the emphasis in variation, SSEA offers a starting point for further research and a deeper understanding of the causes of multiple-driver responses. However, it does not substitute (but instead complements) existing methods, such as statistical inference or any approach based on CAR. Overall, we view SSEA as an addition to the ‘toolbox’ used to advance research in the study of the effects of climate change on biological systems.

## Methods

### Model species

We evaluated the effect of temperature in combination with food limitation or with reduced salinity on the survival of larval stages of a warm-adapted crab (*H. sanguineus*) and a cold-adapted competitor (*Carcinus maenas*). Both species coexist in the Atlantic coast of North America, while *H. sanguineus* is invasive in North Europe where *C. maenas* is native^55,60,61^. Larvae of those species are characterised by different lower temperature limits: for *C. maenas*, complete larval development can be achieved at 9 °C^49^, while for *H. sanguineus* the lowest temperature enabling complete larval development is 15 °C^60^. Larvae of both species can develop at 24 °C^54,60^, but there is no information on the upper temperature limit for larval development. Survival of larvae of *C. maenas* responds antagonistically to increased temperature (range 15–24 °C) and reduced salinity (salinity range: 20–32.5 PSU with control conditions defined at 15 °C and 32.5 PSU^27^. Larvae of *H. sanguineus* from populations of N. America show a similar pattern with increased temperature leading to higher tolerance of low salinity^60^. However, because 15 °C is at the limit of tolerance, and higher survival is achieved in the range 18–24 °C, such pattern would qualify as synergistic or additive (as driven by the combination of reduced temperature and salinity). Because *C. maenas* and *H. sanguineus* are competitors^40,41^, it is important to compare the responses of larvae produced by populations in sympatry.

### Experimental design

We exposed first-stage larvae of *H. sanguineus* and *C. maenas* to combinations of temperature, food limitation and salinity treatments (Supplementary Fig. 6). For both species, there were five replicate units for each treatment combination, consisting of a group of 10–11 larvae per replicate. In addition, the experiment was repeated three times, with larvae from three different females per species; larvae of the different females were kept in separate glasses in order to tease apart the effect of female of origin from that of the environmental factors.

There were four temperature treatments, comprising three treatments (T_1_–T_3_) of constant or gentle temperature variation and a heatwave condition (T_4_). Treatment-1 (T_1_) consisted in keeping the larvae at ‘constant-low temperature’ (16.5 °C) corresponding to the average temperature experienced by larvae of treatment-2 (T_2_), where temperature increased gently. T_2_ represented a ‘baseline scenario’ of spring conditions; T_1_ acted as the check of T_2_, as both shared the same average temperature but differed in the degree and nature of the variation. Larvae in T_2_ were kept under gentle temperature increase for over 6 days (from 15 to 18 °C with increases of 0.5 °C per day); such time period covers the duration of development of first-stage larvae of both species. Treatment-3 (T_3_) consisted in keeping larvae under ‘constant high temperature’ (17.5 °C) that also corresponded to the average temperature experienced by larvae kept under treatment-4 (T_4_). In T_4_, we simulated a scenario of a marine heatwave occurring during spring or early summer when the temperature increases. Larvae exposed to T_4_ (heatwave treatment) were kept at 15 °C for 24 h and then the temperature was increased to 18 °C in a step (within 24 h) and such temperature was maintained until the end of the experiment. Treatments T_2_ and T_3_ help to test the effect of heatwave simulated for T_4_. T_2_ controls for effects of smooth variations in temperature (T_2_ and T_4_ shared the same initial and final temperatures, 15 and 18 °C but differed in the increase rate); T_3_ controls for the effect of the average temperature on larval responses.

For each of the four temperature treatments, we assigned larvae to three groups following a factorial design. Group 1 was reared under optimal conditions of salinity and food: in natural seawater (salinity = 32 PSU), and permanent access to food. Group 2 (low salinity treatment) was reared at salinity = 20 PSU under permanent access to food. Group 3 (food limitation treatment) was reared in natural seawater under limited access to food. Limited access to food followed recent findings showing that crustacean larvae are capable of tolerating food limitation as long as they have access to food for 4–6 h per day^54,62^. Overall, there were 180 replicate units per species, i.e. three repetitions (larvae from three different females) x 4 temperature treatments x 15 replicate units per temperature treatment (five replicates assigned to each of the three food-salinity treatments).

### Experimental procedures

Experiments were carried out with natural UV-treated filtered (0.2 μm) seawater (salinity = 32.5 PSU) in 60 ml glass containers. The temperature was controlled in automated, fully programmable incubators; salinity was controlled by diluting seawater with appropriate amounts of freshwater and by adjusting salinity with a salinometer (WTW). Larvae were fed with freshly hatched *Artemia* sp. nauplii (density ~5 nauplii ml^−1^). For the treatment of food limitation, larvae were fed for 6 h per day between 10 and 16 h. Every day, water and food were changed and larvae were checked for moults or mortality (dead individuals were removed from cultures). We recorded survival every day but here present data on the proportion of larvae reaching the second stage. We analysed survival proportions after logarithmic transformation in order to test for the multiplicative model^10^, but also after logistic transformation which tends to ensure better residuals^63^. Graphical plots are given in the raw and logarithmic scale.

### Data analysis

Analyses were carried out for each species separately, and the effects of food limitation or salinity were evaluated in separate tests. Each test followed a four-way factorial design with temperature regime, temperature level, food level (or salinity) and female of origin as fixed factors. Female of origin was considered as fixed because there were *n* = 3 repetitions of the experiment^64^. Tests were carried out following backwards model selection^65^ based on the adjusted Akaike information criteria in R^65,66^ using mixed modelling (package nlme^67^). Model selection was carried out sequentially by comparing models of different complexity. If the simple model had the lowest AICc, such model was retained. If the complex model had the lowest AICc, the selection was carried out as follows: (1) if ∆AICc > 3 then the complex model was retained; (2) if ∆AICc < 3 models were compared through a likelihood ratio test: if *p* < 0.05, the complex model was retained; otherwise, the simple model was retained.

After model selection, we used two criteria to assign a treatment effect as ‘heatwave effect’: First, there should be a significant difference between the treatment simulating the heatwave (T_4_) vs. the control for the effects of a gradual temperature increase (T_2_) and the one characterised by the average temperature experienced during the heatwave (T_3_). Thus, a heatwave effect should be detected as an additive effect or a form of interactive effect between two factors where T_4_ differs from any other treatments. Second, larval mortality had to occur primarily after the temperature increase was applied at the time course of the experiment. We, therefore, calculated the proportion of larvae dying after the temperature increased for the case that a treatment effect would match the first criteria; we confirmed that such criteria was also met (Supplementary Table 1).

SSEA calculations for Fig. 6 were carried out using treatment T_1_ as reference (i.e. low and constant temperature), separately for larvae from each female and each of the three food-salinity treatments; note that each food-salinity treatment had its own T_1_ treatment (Supplementary Fig. 6). Additional SSEA calculations were made for Supplementary Fig. 10 and details are given in the supplement. The data were available in a database^68^.

### Reporting summary

Further information on research design is available in the Nature Research Reporting Summary linked to this article.

## Supplementary information

Supplementary Information

Description of Supplementary Files

Supplementary Data 1

Reporting Summary

## Data Availability

Data of the experiment simulating heatwaves will be available in the PANGEA portal (ref. ^68^). Data corresponding to Fig. 3 is provided in a file. R code is available in the Supplement.
