# Supplementary material for: A state-space approach to understand responses of organisms, populations and communities to multiple environmental drivers
Source: Commun Biol. 2021 Sep 30;4:1142. doi: 10.1038/s42003-021-02585-1 (PMC8484576; doi:10.1038/s42003-021-02585-1)
Supplement: Supplementary file 1 — Supplementary Information [file 42003_2021_2585_MOESM1_ESM.pdf]

A state-space approach to understand responses of organisms, populations and communities to multiple drivers

SUPPLEMENTARY INFORMATION

Supplementary Note 1: responses as a bivariate smooth function subject to a Taylor expansion

The aim of this section is to show that $D_1(a)$, $D_2(b)$ and $G(a,b)$ are values given by linearly independent polynomials and hence they can be used to construct a geometric representation of the response $R\ (a,b)$. Recall that the state variables represent average responses quantified at stressor levels, a and b (for environmental drivers E_1 and E_2 respectively), defined in a factorial experiment (Supplementary Figure 1). Our objective is to obtain:

$$D_1(a) = f_1 \quad (1a)$$

$$D_2(b) = f_2 \quad (1b)$$

$$R(a,b) - D_1(a) - D_2(b) - R(0,0) = g \quad (1c)$$

First, we model the response, $R(E_1, E_2)$, as bivariate function (Supplementary Figure 1). We then approximate by a polynomial Taylor expansion around the control treatment to obtain:

$$R(E_1, E_2) = f_1(E_1) + f_2(E_2) + g(E_1^m \cdot E_2^n) \quad (2)$$

In (1) the response is driven two functions, $f_1 = f(E_1)$ and $f_2 = f(E_2)$, describing the additive contribution of each single stressor, and a third function, $g = g(E_1^m \cdot E_2^n)$, describing additional contributions. Each function (f_1, f_2, g) is a polynomial, i.e. a sum of linear and higher order terms. The superscripts n and m are integer exponents.

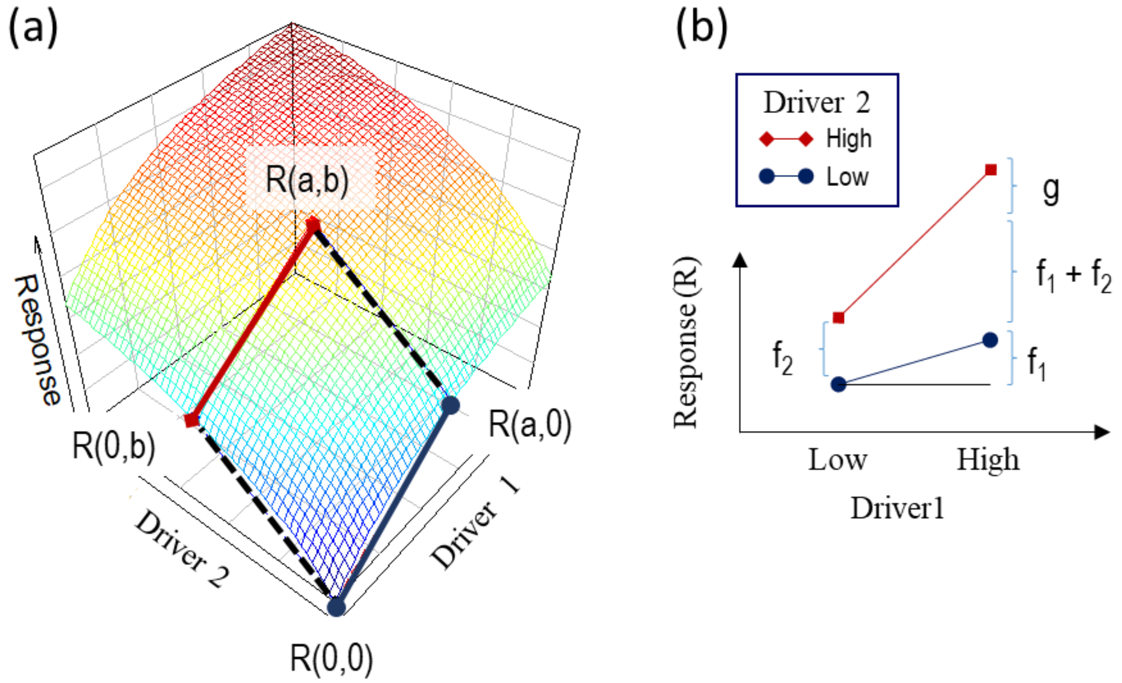

Supplementary Figure 1. (a) A response (R) to two environmental drivers viewed as a smooth bivariate function. Points define treatment combinations applied at specific stressor levels. The control condition, R(0,0), is defined as the origin of the coordinate system (stressor levels = 0 and stress response = 0). Lines define differences among responses at such treatment combinations. (b) Projection of the response in the interaction plot. After the Taylor expansion (see text), differences among treatment combinations can be identified as independent components (f_1, f_2, g) of the response *R(a,b)*.

We perform a Taylor expansion of $R(E_1, E_2)$. A Taylor expansion of the multivariable function, R(x,y), around a point (a,b) is as follows:

$$R(x,y) = R(a,b) + R_x(a,b) \cdot (x-a) + R_y(a,b) \cdot (y-b) +$$

$$0.5 \cdot \left[R_{xx}(a,b) \cdot (x-a)^2 + R_{yy}(a,b) \cdot (y-b)^2 + 2 \cdot R_{xy}(a,b) \cdot (x-a) \cdot (y-b)\right] + O(x,y)$$

R_x and R_y are the first order partial derivatives of R with respect to x and y respectively, R_{xx} , R_{yy} and R_{xy} are the second order partial derivatives. O(x,y) represent higher other terms containing higher order derivatives.

We then expand the function around the point defined at *a=0* and *b=0:*

$$R(x,y) = R(0,0) + R_x(0,0) \cdot x + R_y(0,0) \cdot y + 0.5 \cdot \left[R_{xx}(0,0) \cdot x^2 + R_{yy}(0,0) \cdot y^2 + 2 \cdot R_{xy}(0,0) \cdot x \cdot y\right] + O(x,y)$$

Rearranging terms we have:

$$R(x,y) = R(0,0) + R_x(0,0) \cdot x + 0.5 \cdot R_{xx}(0,0) \cdot x^2 + O(x^m) + R_y(0,0) \cdot y + 0.5 \cdot R_{yy}(0,0) \cdot y^2 + O(y^n) + R_{xy}(0,0) \cdot x \cdot y + O(x^m y^n)$$

We obtain expression 1 by taking the following definitions:

$$f_1(x) = R_x(0,0) \cdot x + 0.5 \cdot R_{xx}(0,0) \cdot x^2 + O(x)$$

$$f_2(y) = R_y(0,0) \cdot y + 0.5 \cdot R_{yy}(0,0) \cdot y^2 + O(y)$$

$$g(x \cdot y) = R_{xy}(0,0) \cdot x \cdot y + O(x^m \cdot y^n)$$

For example, in the case of a third order expansion we have:

$$f_1(x) = R_x(0,0) \cdot x + 0.5 \cdot R_{xx}(0,0) \cdot x^2 + \frac{1}{6} R_{xxx}(0,0) \cdot x^3$$

$$f_2(y) = R_y(0,0) \cdot y + 0.5 \cdot R_{yy}(0,0) \cdot y^2 + \frac{1}{6} R_{yyy}(0,0) \cdot y^3$$

$$g(x \cdot y) = R_{xy}(0,0) \cdot x \cdot y + \frac{3}{6} [R_{xxy}(0,0) \cdot x^2 \cdot y + R_{xyy}(0,0) \cdot y^2 \cdot x]$$

We now return to the factorial design with four treatment combinations and derive the following relationships:

$$D_1(a) = R(a,0) - R(0,0) = f_1(a) - f_1(0) \text{ (3a)}$$

$$D_2(b) = R(0,b) - R(0,0) = f_2(b) - f_2(0) \text{ (3b)}$$

$$R(a,b) - R(0,b) = g(ab) - g(0) + R(a,0) - R(0,0) \text{ (3c)}$$

Equations (3a) and (3b) quantify the contribution of each individual stressor (their sum is the predicted additive effect); eq. 2c quantifies the departure from an additive response, because when *g(ab)-g(0) =0* we obtain an equality to the additive response.

By setting the origin at the coordinate system to *f(0)=0* and *f(0)=0* and *g(0)=0*, we obtain *R(0,0)=0* and the following relationships.

$$D_1(a) = f_1$$

$$D_2(b) = f_2$$

$$R(a,b) - D_1(a) - D_2(b) - R(0,0) = g$$

We therefore find that the differences shown in equation (3) correspond to the values of the linearly independent polynomials at the points *a* and *b*.

Supplementary Note 2: Extended analysis to produce Figures 3

Supplementary figure 2 (next page) extends the analysis shown in Figure 3 of the main text based on the data was obtained by Durrant et al (2013) on the effect of temperature and pH on growth of a marine bryozoan. Data extracted from Figure 2b, through the freeware www.graphreader.com accessed on 16.11.2020 and stores in datafile Durrant-fig2b.xlsx.

The experiment consisted of bryozoan colonies (labelled A-M) to 2 temperatures (control and increased temperature) and 3 pH levels (control, 7.8 and 7.6). In Supplementary Figure 2, panel **(a)** reproduces the mean values plotted in Figure 2b of Durrant et al. (2013); values corresponding to the control conditions, increased temperature and pH=7.8 are plotted in Figure 3a of the main text. Panels **(b,d)** show the state-space representation for the combined effect of increased temperature and reduced pH=7.8 (as in Fig. 3b,c of main text); panels **(c,e)** show a similar representation (based on the same control treatment + treatment of increased temperature) but with the data from pH=7.6.

Comparisons between e.g. panels **(d)** and **(e)** provide an example for visualization of the importance of stressor intensity (here pH values). We can see an important level of variability in how colonies respond to pH. For instance, colony L shows a synergistic effect of increased temperature and moderately low pH but this pattern switches to antagonistic at the lowest tested pH; by contrast, colonies C and K show consistent antagonistic responses at both levels of reduced pH.

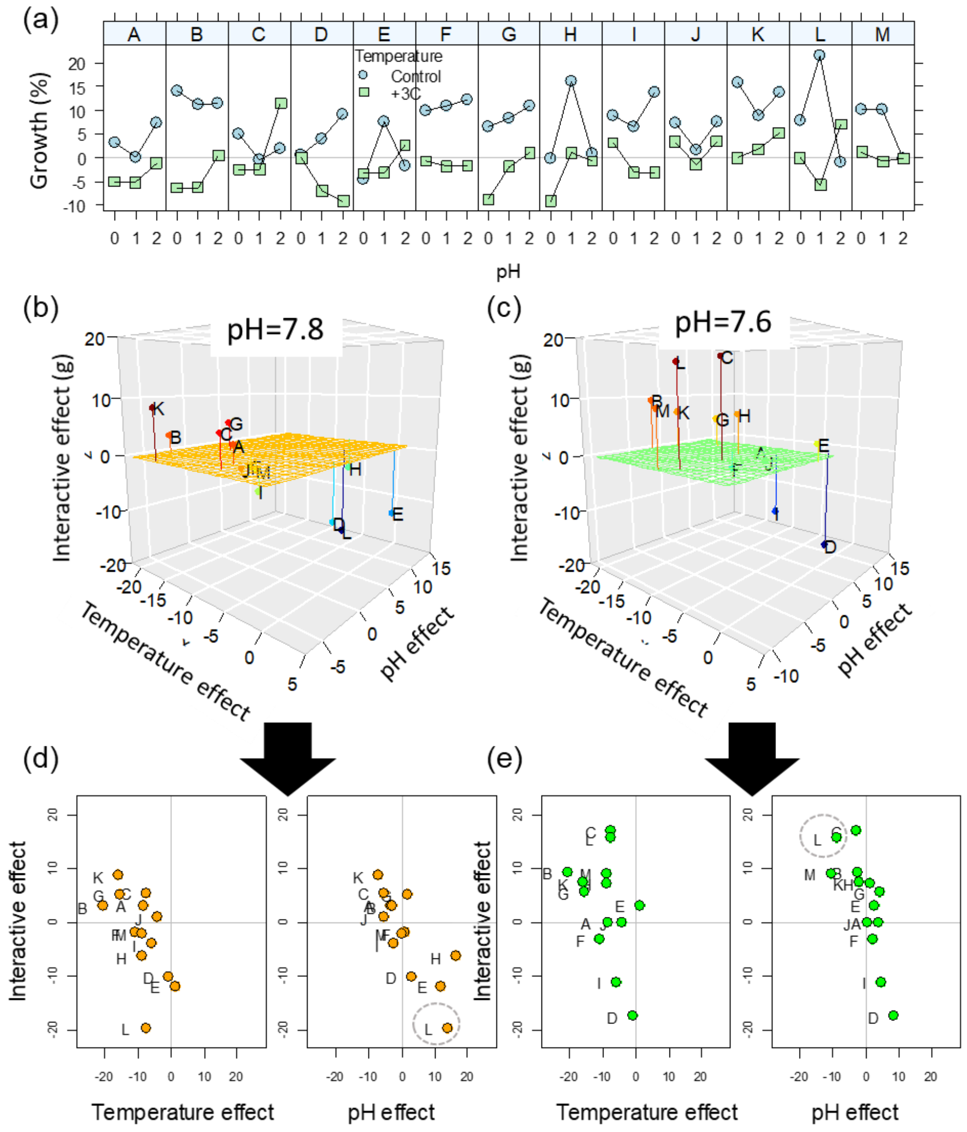

Supplementary Figure 2. Summary of effects of increased temperature and reduced pH on growth of colonies (A-M) of a marine bryozoan. **(a)** Interaction plots; **(b-e)** space-state representation. See text in previous page for further explanations.

Reference: Durrant, HMS, Clark GF, Dworjanyn SA, Byrne M, Johnston EL (2013). Seasonal variation in the effects of ocean warming and acidification on a native bryozoan, *Celleporaria nodulosa*. Marine Biology 160: 1903-1911.

Supplementary algorithm, 1: R code for simulations used to produce Figures 3 and Supplementary figure 2

```r
########################CODE IN RSTUDIO ################################
#=================================================================================
# State-space representation of data from Durrant et al (2013)
# Effect of pH and temperature on colony growth: each colony represents a different
genotype
# This code produces plots in figure S2

# Libraries needed
library(readxl)
library(lattice)
library(gridExtra)
library(latticeExtra)

library(plot3D)
# Data ===========================================================================
Durrant_fig2b <- read_excel("Durrant-fig2b.xlsx")
df<-Durrant_fig2b

# Data:  temperature x pH interaction for colonies named as A to M
# 2 temperatures: Control and +3C =P
# 3pH levels: control=0, 7.8=1 and 7.6=2

# Interaction plot: Figure 3a=====================================================

mypanelt = function( x,y,...) {
  panel.abline( h=0, col = "grey")
  panel.xyplot( x,y,...)
}
mykey<-list(title= "Temperature",cex.title = 0.8,
            x=0.3, y=0.90,
            points= list(col="black", fill=c("light blue", "darkseagreen2"), pch=c(
21,22), cex=c(1.0,1.0)),
            text = list(c("Control", "+3C"),cex=0.8))
xyplot(growth~pH|Colony,groups=Temperature, data=df, as.table=TRUE,
       strip=strip.custom(bg="aliceblue"), layout=c(13,1),
       cex=1.2, pch=c(21,22),col="black", fill=c("light blue", "darkseagreen2"), ty
pe=c("p","l"),
       scales=list(alternating=FALSE,x=list(at=c(0,1,2))),
       ylab="Growth %",
       xlim=c(-0.5,2.5),
       panel=mypanelt,
       key = mykey)



#=================================================================================
```

```
# Calculations
# This loop calculates f1,f2 and g for each separate colony

# fT=temperature fph = pH, g= interaction temperature:pH

# Each colony has its own control treatment
spst<-NULL
for (i in c("A","B","C","D","E","F","G","H","I","J","K","L","M")){
    dft<-subset(df, Colony==i)
  control<-subset(dft,Temperature=="c" & pH==0) # control=R(0,0)
  dft$cc<-dft$growth-control$growth # cc=R(a,b)-R(0,0) for any a and b.

# Storing fT, fph and interaction data in data frame
  tstsp<-data.frame(fT=rep(dft$cc[4],2),fph=c(dft$cc[2:3]),ms=c(dft$cc[5:6]))
  tstsp$add<-tstsp$fT+tstsp$fph # Calculate A(a,b): additive effect
  tstsp$int<-tstsp$ms-tstsp$add # Calculate g:
  tstsp$colony<-i # storing colony name
  tstsp$ph<-c(7.8,7.6) # storing ph levels for different plots
  spst<-rbind(spst,tstsp) # accumulating data over colonies
  }


# Graphs ==========================================================================
# Data are subsetted to produce plots of figures 3a and 3b in paper
spst78<-subset(spst,ph==7.8)
spst76<-subset(spst,ph==7.6)

# Figure 3b: 3D representation: x=fT, y=fph, z=interactive effect

# i) Create vertical lines from data points projected on a plane at z=0
# to data points in the 3D space
xplane=seq(min(spst78$fT), max(spst78$fT))
yplane=seq(min(spst78$fph),max(spst78$fph))
xy<-expand.grid(x=xplane, y=yplane)
zplane<-matrix(rep(0,nrow(xy)),nrow=22,ncol=24)
zpoints<-rep(0,13)

# ii) graph

scatter3D(spst78$fT,spst78$fph,spst78$int,
          phi=10, bty ="g", zlim=c(-20,20),ylim=c(-7,15),xlim=c(-22,5),
          pch=20, cex=1.2, colkey=FALSE, ticktype="detailed",
             # projections calculated in (i) added with surf function
          surf= list(x=xplane,y=yplane,z=zplane, facets=NA, fit=zpoints))
# iii) add text labels to graph
text3D(spst78$fT,spst78$fph,spst78$int,
       labels=spst78$colony, colkey=FALSE, add=TRUE)


# Figure 3d: 2D projections

mypanel = function( x,y,...) {
```

```
  panel.abline( h=0, v=0, col = "grey")
  panel.text(x, y, labels=spst78$colony, offset=1,pos=2, cex=0.7)
  panel.xyplot( x,y,...)
}

gT<-xyplot(int~fT, ylab=list(label="Interactive effect", cex=0.8),
           xlab=list(label="Temperature effect",cex=0.8),xlim=c(-25,5),
           scales=list(x=list(cex=0.6), y=list(cex=0.6)),
           pch=c(21), cex=1.0, col="black", fill="orange",
           strip=strip.custom(bg="aliceblue"), panel=mypanel,
           data=spst78)

gph<-xyplot(int~fph, ylab=list(label="Interactive effect", cex=0.8),
           xlab=list(label="pH effect",cex=0.8),xlim=c(-10,20),
           scales=list(x=list(cex=0.6), y=list(cex=0.6)),
           pch=c(21), cex=1.0, col="black", fill="orange",
           strip=strip.custom(bg="aliceblue"),
           panel=mypanel,
           data=spst78)

tot<-c(gT,gph, y.same=FALSE,x.same=FALSE, layout=c(2,1))
update(tot, xlab=c("Temperature effect", "pH effect"), ylab="Interactive effect",
       ylim=c(-20.5,20.5), xlim=c(-25,25))

# Figure 3c: 3D representation

xplane2=seq(min(spst76$fT), max(spst76$fT))
yplane2=seq(min(spst76$fph),max(spst76$fph))
xy<-expand.grid(x=xplane2, y=yplane2)
zplane2<-matrix(rep(0,nrow(xy)),nrow=22,ncol=19)
zpoints2<-rep(0,13)

scatter3D(spst76$fT,spst76$fph,spst76$int,

          phi=10,bty ="g", zlim=c(-20,20),ylim=c(-11,15),xlim=c(-22,5),
          pch=20, cex=1.2,colkey=FALSE, ticktype="detailed",
          surf= list(x=x plane2,y=y plane2,z=z plane2, facets=NA, fit=zpoints2))

text3D(spst76$fT,spst76$fph,spst76$int,
       labels=spst76$colony, colkey=FALSE, add=TRUE)


# Figure 3e: 2D projections
mypanel2 = function( x,y,...) {
  panel.abline( h=0, v=0, col = "grey")
  panel.text(x, y, labels=spst76$colony, offset=1,pos=2, cex=0.7)
  panel.xyplot( x,y,...)
}
gT2<-xyplot(int~fT, ylab=list(label="Interactive effect", cex=0.8),
           xlab=list(label="Temperature effect",cex=0.8),xlim=c(-25,5),
           scales=list(x=list(cex=0.6), y=list(cex=0.6)),
```

```
           pch=c(21), cex=1.0, col="black", fill="green",
           panel=mypanel2,
           data=spst76)

gph2<-xyplot(int~fph, ylab=list(label="Interactive effect", cex=0.8),
            xlab=list(label="pH effect",cex=0.8),xlim=c(-10,20),
            scales=list(x=list(cex=0.6), y=list(cex=0.6)),
            pch=c(21), cex=1.0, col="black", fill="green",
            panel=mypanel2,
            data=spst76)

tot2<-c(gT2,gph2, y.same=FALSE,x.same=FALSE, layout=c(2,1))
update(tot2, xlab=c("Temperature effect", "pH effect"), ylab="Interactive effect",
       ylim=c(-20.5,20.5),xlim=c(-25,25))

######## End of code Figure 3###################################################
```

Supplementary algorithm 2: R code for simulations used to produce Figure 4

```
# Figure 4: Synergistic effect increasing through time ################
library(lattice)
library(gridExtra)
# Generation of data===================================================
# Temperature, habitat and time with arbitrary values
foo<-data.frame(expand.grid(Temp= c(10,20), Habitat=c(20,30),
                            Time=seq(1,10,1)))
# Performance data
# Good habitat: performance (dd) increases linearly with temperature (T) and is con
stant through time: dd= c1+b1*T
# Poor habitat: performance increases linearly with temperature:
    dd=c1+f(time)*T
# But the rate of increase is driven by time, following a logistic function:f(time)
= b1+1/(1+exp(5-time))

b1=0.1
c1=10
foo$dd<-ifelse( foo$Habitat==20,c1+foo$Temp*b1,
               c1+foo$Temp*(b1+1/1+exp(-foo$Time+5)) )


# Figures =============================================================
# Figure 4a
mykey2<-list(title= "Habitat",cex.title = 1.2,
      x=0.05, y=0.95, points= list(col="black",
      fill=c("light blue", "light green"),pch=c(21,22),cex=c(1.0,1.0)),
       text = list(c("Good", "Poor"),cex=1.0))
xyplot(dd~factor(Temp)|factor(Time), groups=factor(Habitat),
```

```
       ylab=list(label="Performance", cex=1.2),
       xlab=list(label="Temperature",cex=1.2),
       scales=list(x=list(cex=1.2), y=list(cex=1.0), relation="free"),
       ylim=c(9,40),
       pch=c(21,22), cex=1.2, col="black",
       fill=c("light blue", "light green"),
       data=foo,  key=mykey2,type ="b",lty=1:2, layout=c(2,5),
       as.table=TRUE,
       strip=strip.custom(bg="aliceblue"))
```

```
#Figure 4b
mykey3<-list(title= "Habitat",cex.title = 1.2,
             x=0.05, y=0.90,
             points= list(col="black", fill=c("light blue", "light green"), pch=c(2
1,22), cex=c(1.0,1.0)),
             text = list(c("Good", "Poor"),cex=1.0))
xyplot(dd~Time|factor(Temp), groups=factor(Habitat),  ylab=list(label="Performance"
, cex=1.2),
       xlab=list(label="Time",cex=1.2),
       scales=list(x=list(cex=1.2), y=list(cex=1.0), relation="free"),

       ylim=c(9,40),
       pch=c(21,22), cex=1.2, col="black",
       fill=c("light blue", "light green"),
       data=foo,  key=mykey3,type ="b",lty=1:2, layout=c(2,1), as.table=TRUE,
       strip=strip.custom(bg="aliceblue"))
```

```
# Figure 4c Space-state representation
# Calculations are as in the Figure 3 but "looping" through each time step
# At each time step there is a value of the control and of each remaining    treatm
ent combination

fstsp<-NULL
for (i in 1:10) {
  tfoo<-subset(foo, Time==i) # select a time step
  controlt=subset(tfoo,Temp==10 & Habitat==20) # Assign control
  tfoo$cc<-tfoo$dd-controlt$dd # cc=R(a,b)-R(0,0) for any a and b.
  tstsp<-data.frame(f1=rep(tfoo$cc[3]),f2=c(tfoo$cc[2]),ms=c(tfoo$cc[4])) # Storing
f1 and f2 in data frame; also storing data for interactive effect
  tstsp$add<-tstsp$f1+tstsp$f2 # Calculate A(a,b)
  tstsp$int<-tstsp$ms-tstsp$add # Calculate g
  fstsp<-rbind(fstsp,tstsp) # accumulate data over time
}
spt<-fstsp # securing data in separate data frame
spt$Time<-seq(1,10,1) # Adding time steps

# Plotting Figure 4c: 2D representation ignoring f2 because it is constant
g3<-xyplot(int~f1|"Space state",
```

```
            ylab=list(label="Interactive effect", cex=1.2),
            xlab=list(label="Temperature effect",cex=1.2),
            scales=list(x=list(cex=1.0), y=list(cex=1.0)),
            pch=c(21), cex=1.2, col="black", fill="dark cyan",
            strip=strip.custom(bg="aliceblue"),
            data=spt)
# Changes in the magnitude of the interactive effect through time
g4<-xyplot(int~Time|"Time evolution",
            ylab=list(label="Interactive effect", cex=1.2),
            xlab=list(label="Time",cex=1.2),
            scales=list(x=list(cex=1.0), y=list(cex=1.0)),
            pch=c(21), cex=1.2, col="black", fill="dark cyan",
            strip=strip.custom(bg="aliceblue"),
            data=spt)

grid.arrange(g3,g4)

##### End of code for Figure 4 ##############################################
```

Supplementary Note 3: Dealing with artefacts in interaction terms: worked example

In this section, we show an example of an artefact, occurring when a response is additive, and with types of responses that are similar to the example given in Fig. 3 of the main manuscript. We model 1000 virtual factorial experiments where the effects of two environmental factors are evaluated at two levels. A total of n-samples are used to estimate the treatment means; samples are drawn from a Gaussian distribution with constant standard deviation = σ.

We present three cases: Case (1) Additive, i.e. $R(a,b) = R(a,0)+R(0,b)$. The response varies among experiments but it is always additive with constant n and σ. Case (2): Interactive fixed, $R(a,b) = R(a,0)+R(0,b)+10$. The response is as in case-1 but the 10 units of effect are added to $R(a,b)$ in order to obtain an interactive effect in each of the experiments. Case (3) Interactive correlated, $R(a,b) = -R(a,0)(1-\gamma)+R(0,b)+10$. The response is interactive with g depending on f_1.

In such situation (Supplementary Figure 3), the SSEA shows an apparent relationship between g and each f_i. Several repetitions of the same simulation show that the correlation between g and each f_i is low (~0.2) and significant only when a large number of experiments is modelled. In addition, the correlation decreases if the number of replicate samples increases or the standard deviation decreases. Simulations adding a significant interacting effect also show the apparent relationship (Supplementary Figure 4). Here, we propose two methods to deal with an apparent pattern after a preliminary step.

Preliminary step: In order to speed up calculations one can estimate f_1, f_2, and g as follows: First, recode the factors as two dummy variables, X_1 and X_2 based on (0,1) coding. Treatment combinations are coded as follows: Control: $X_1 = 0$, X_2=0, Factor-1: $X_1 = 1$, X_2=0, Factor-2: $X_1 = 0$, X_2=1, Interaction: $X_1 = 1$, X_2=1. Then, fit a linear model to the data: in R this would be as follows: model1<-lm(Response~X1*X2, data=mydata). For each replicate experiment, the parameter estimates corresponding to X_1, X_2 and X_1:X_2 will give f_1, f_2, and g respectively. One can see in our simulated example that the "by hand" approach of calculating means and then subtracting the appropriate terms is equivalent to the method proposed here.

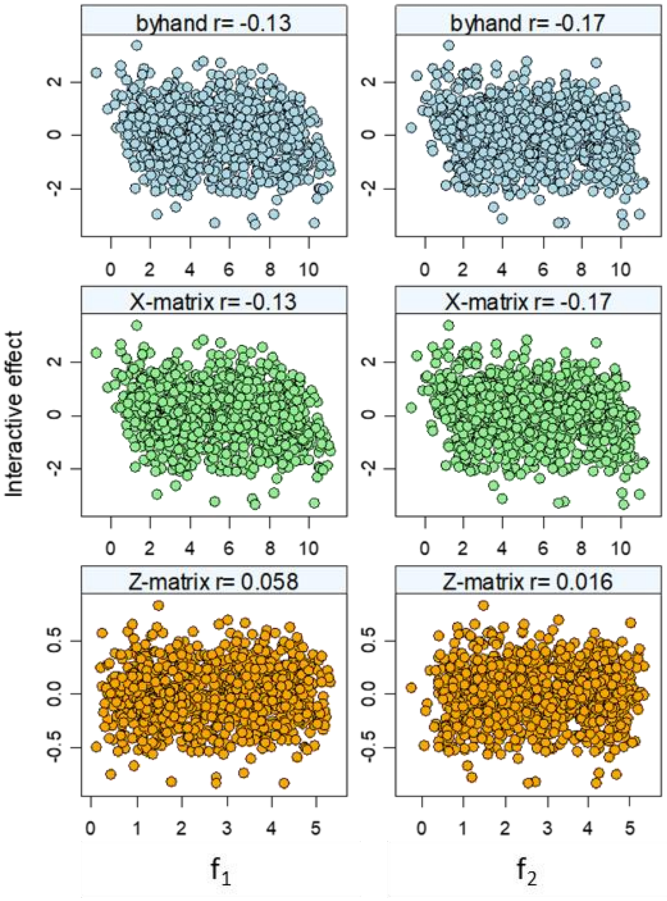

Supplementary Figure 3. Biplots of SSEA representation for a simulation of 1000 experiments showing an additive response of different magnitude. Three representations are shown: (1) "By hand" (blue circles): by first calculating means per treatment combinations and then applying equations given in text. (2) X-matrix (green circles): fitting a linear model to the raw data using dummy coding (0,1) for the factors, so that the reference is coded as (0,0); this representation is equivalent to "by hand". (3) Z-matrix (orange circles): fitting a linear model to the raw data using zero-sum dummy coding (-1,1) so that the reference is coded as (-1,-1). Correlations are provided between each f_i and the interactive effect.

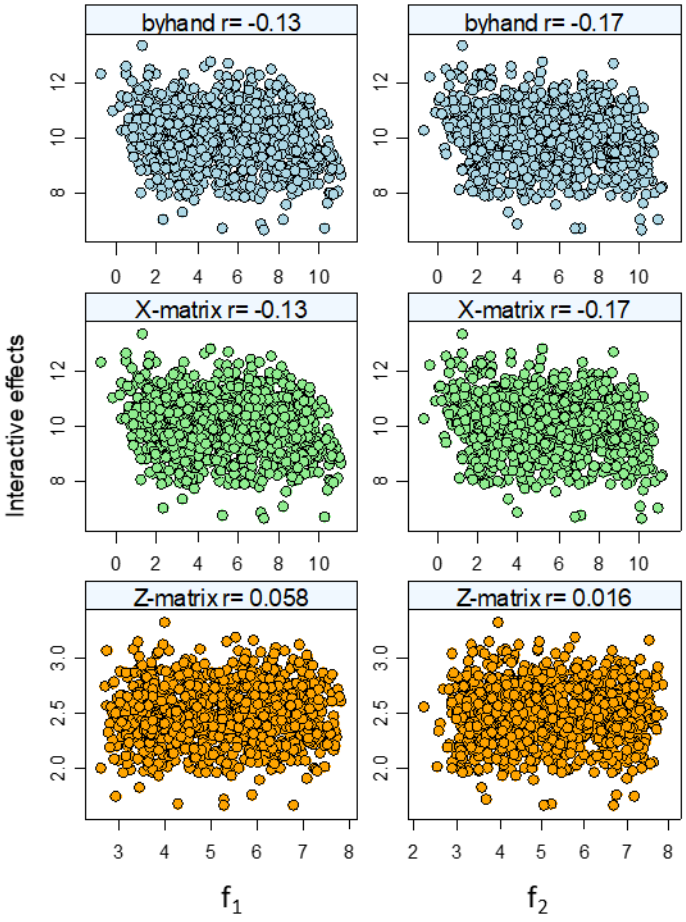

Supplementary Figure 4. Biplots of SSEA representation for a simulation of 1000 experiments showing an interactive response of fixed magnitude while the additive components are as in Fig. Supplementary Figure 3. Three representations are shown: (1) “By hand” (blue circles): by first calculating means per treatment combinations and then applying equations given in text. (2) X-matrix (green circles): fitting a linear model to the raw data using dummy coding (0,1) for the factors, so that the reference is coded as (0,0); this representation is equivalent to “by hand”. (3) Z-matrix (orange circles): fitting a linear model to the raw data suing zero-sum dummy coding (-1,1) so that the reference is coded as (-1,-1). Correlations are provided between each f_i and the interactive effect.

Supplementary Method 1: Check significance of interaction terms. Based on the linear model fitted in the preliminary step, we can compute the proportion of experiments giving a significant interaction term. Our simulations show that under the additive model, the proportion of experiments giving significant effects at critical level alpha = 0.05 is 5% in agreement with expectations. Therefore, under a similar

scenario one would conclude that any pattern in *g* is an artefact, as experiments do not show evidence of following an interactive pattern.

Supplementary Method 2: Do the SSEA representation based on a zero sum design matrix. Recode factors as dummy variables Z_1 and Z_2 based on (-1,1) coding. Treatment combinations are coded as follows: Control: Z_1 = -1, Z_2=-1, Factor-1: Z_1 = 1, Z_2=-1, Factor-2: Z_1 = -1, Z_2=1, Interaction: Z_1 = 1, X_2=1. Alternatively, create a contrast vector based on zero-sum coding. Fit a model: in R, an appropriate code would be model2<-lm(Response~Z1*Z2, data=mydata). For each replicate experiment, the parameter estimates corresponding to Z_1, Z_2 and Z_1:Z_2 will give a new SSEA representation z_1, z_2, and *w* (for the interaction) respectively. If you use zero-sum contract vectors, you might need to multiply the output of z_1, z_2 by -1 because the coding is (1,-1) instead of (-1,1). Our simulations show that the centred representation removes artefacts but leaves patterns that are real (Supplementary Figure 5).

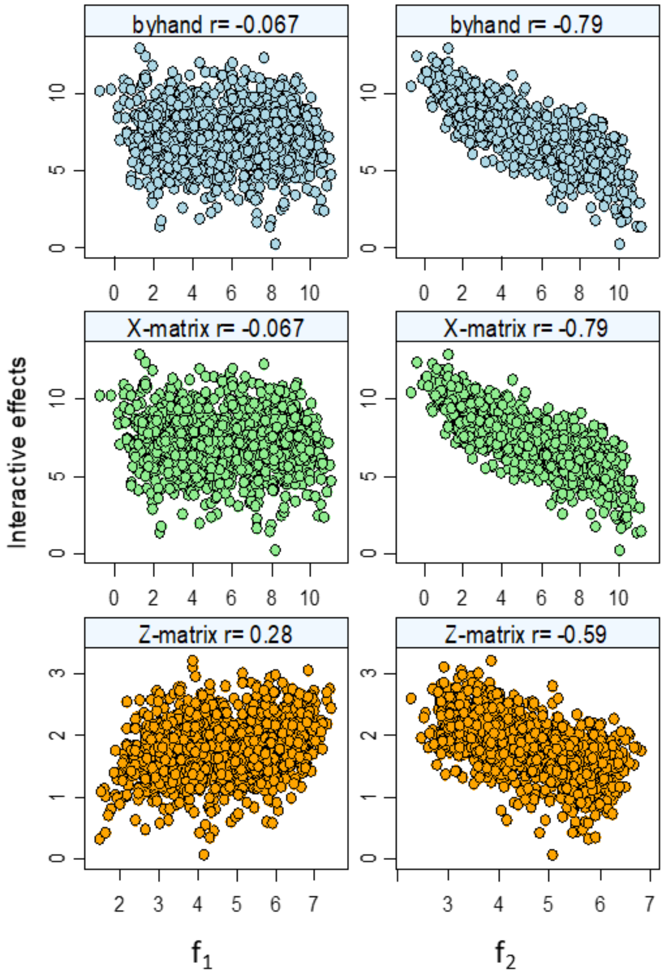

Supplementary Figure 5. Biplots of SSEA representation for a simulation of 1000 experiments showing an interactive response correlated to the magnitude of one of the main effect component. The magnitudes of the additive components are as in Supplementary Figure 3. Three representations are shown: (1) “By hand” (blue circles): by first calculating means per treatment combinations and then applying equations given in text. (2) X-matrix (green circles): fitting a linear model to the raw data using dummy coding (0,1) for the factors, so that the reference is coded as (0,0); this representation is equivalent to “by hand”. (3) Z-matrix (orange circles): fitting a linear model to the raw data suing zero-sum dummy coding (-1,1) so that the reference is coded as (-1,-1). Correlations are provided between each fi and the interactive effect.

Supplementary algorithm 3: Code for worked example (Supplementary Note 3)

```
# The following codes provides a guide to remove artefacts for the case of an addit
ive effects.

# Libraries

library(lattice)
library(latticeExtra)
library(gridExtra)


#######################################################################

# Step 1: Generating simulated data

# Each experiment is a factorial 2x2 with responses defined as

# Control = R(00), Single driver: R(a0) and R(0b), Multiple driver: R(ab)
# N = 1000 experiments are simulated, each one with a different result
N=1000
mm<-runif(N,5,10)

# Three different Responses, originated from

# 1) RespAdd; An additive effect:

# 2) RespInt A: fixed interactive effect:

# 3) RespIntB: An interactive effect where the strength of the interaction depends
# on R(a0)-(R00)

mydata<-data.frame(matrix(ncol = 6, nrow = 0))
colnames(mydata)<-c("Exp","D1","D2","RespAdd", "RespInt", "RespIntB")
rr<-4
for (i in 1:N){
  # indices j and k are used to simulate experimental results with different effect
# sizes per experiment
  j=rep(runif(1,1,10),rr*4)
  k=rep(runif(1,1,10),rr*4)


  # coefficient to simulate correlation between f and g
  gama<-rnorm(1,0.5,0.1) # Draw values of gamma from a Gaussian distribution

  # Simulation of a factorial experiment with 2x2 factor combinations
# using notation of paper:
  # Response: R(0,0), R(a,0),R(0,b),R(a,b)
  # Additive pattern
  R00<-rnorm(rr,mm[i],1) # Control
  Ra0<-rnorm(rr,mm[i]+j,1) # Effect of factor 1 = control + an effect of size = j
  R0b<-rnorm(rr,mm[i]+k,1)# Effect of factor 2 (same idea as factor 1)
```

```
  Rab<-rnorm(rr,mm[i]+j+k,1) # Combined effect of factors 1 and 2 as additive
  Rabint<-Rab+10 # Genereting an interactive response by adding a constant


# Generating a response with a correlation
  RabintB= Rab+10-gama*(Ra0-R00)
#Generating data file with: Experiment number, Driver levels and response
  Exp<-rep(i,rr*4)
  D1=rep(c("O","O","a","a"), each=rr)
  D2=rep(c("O","b","O","b"), each=rr)
  RespAdd<-c(R00, Ra0,R0b,Rab)
  RespInt<-c(R00, Ra0,R0b,Rabint)
  RespIntB<-c(R00, Ra0,R0b,RabintB)

  mydatat<-cbind(Exp,D1,D2,RespAdd,RespInt, RespIntB)
  colnames(mydatat)<-c("Exp","D1","D2","RespAdd", "RespInt", "RespIntB")
  mydata<-rbind(mydata,mydatat)
}
mydata$RespAdd<-as.numeric(mydata$RespAdd)
mydata$RespInt<-as.numeric(mydata$RespInt)
mydata$RespIntB<-as.numeric(mydata$RespIntB)

#=============================================================================
# DATA ANALYSIS STARTS HERE

# Add dummy code to data, checking that the coding of driver levels coincide

mydata$X1<-ifelse(mydata$D1=="O",0,1)
mydata$X2<-ifelse(mydata$D2=="O",0,1)
mydata$Z1<-ifelse(mydata$D1=="O",-1,1)
mydata$Z2<-ifelse(mydata$D2=="O",-1,1)

# Run loop to compute f1, f2 and g
addbeta.hats<-matrix(NA,ncol=4,nrow=N)
addzeta.hats<-matrix(NA,ncol=4,nrow=N)
addby.hand<-matrix(NA,ncol=3,nrow=N) # “by hand” calculation for comparison

intbeta.hats<-matrix(NA,ncol=4,nrow=N)
intzeta.hats<-matrix(NA,ncol=4,nrow=N)
intby.hand<-matrix(NA,ncol=3,nrow=N)

intBbeta.hats<-matrix(NA,ncol=4,nrow=N)
intBzeta.hats<-matrix(NA,ncol=4,nrow=N)
intBby.hand<-matrix(NA,ncol=3,nrow=N)

p1add<-p2add<-p1int<-p2int<-p1intB<-p2intB<-matrix(NA,ncol=1,nrow=N)
```

```
# loop starts here-----------------------------------------------------

for (j in 1:N){
  df<-subset(mydata, Exp==j)
  # Parameters are calculated using linear model fitting

# Model fiting with (0,1) coding: uncentered factors
m1<-lm(RespAdd~X1*X2, data=df)

# Model fiting with (-1,1) coding: centered factors
m2<-lm(RespAdd~Z1*Z2, data=df)

mint1<-lm(RespInt~X1*X2, data=df)
mint2<-lm(RespInt~Z1*Z2, data=df)

mintB1<-lm(RespIntB~X1*X2, data=df)
mintB2<-lm(RespIntB~Z1*Z2, data=df)


# Storing p-values of interaction term

 p1add[j,]<-summary(m1)$coefficients[4,4]
 p2add[j,]<-summary(m2)$coefficients[4,4]

 p1int[j,]<-summary(mint1)$coefficients[4,4]
 p2int[j,]<-summary(mint2)$coefficients[4,4]

 p1intB[j,]<-summary(mintB1)$coefficients[4,4]
 p2intB[j,]<-summary(mintB2)$coefficients[4,4]

  # By hand calculations
  medias.add<-aggregate(RespAdd~X1+X2, FUN=mean, data=df)
  medias.int<-aggregate(RespInt~X1+X2, FUN=mean, data=df)
  medias.intB<-aggregate(RespIntB~X1+X2, FUN=mean, data=df)

  f1add<- medias.add[2,3]-medias.add[1,3]
  f2add<- medias.add[3,3]-medias.add[1,3]
  Aaad<-f1add+f2add
  gadd<-medias.add[4,3]-Aaad-medias.add[1,3]
  byhandadd<-c(f1add,f2add,gadd)

  f1int<- medias.int[2,3]-medias.int[1,3]
  f2int<- medias.int[3,3]-medias.int[1,3]
  Aint<-f1int+f2int
  gint<-medias.int[4,3]-Aint-medias.int[1,3]
  byhandint<-c(f1int,f2int,gint)

  f1intB<- medias.intB[2,3]-medias.intB[1,3]
  f2intB<- medias.intB[3,3]-medias.intB[1,3]
  AintB<-f1intB+f2intB
```

```
  gintB<-medias.intB[4,3]-AintB-medias.intB[1,3]
  byhandintB<-c(f1intB,f2intB,gintB)

  # Parameter estimates are stored
  # Additive case
  addbeta.hats[j,] = m1$coef
  addzeta.hats[j,] = m2$coef
  addby.hand[j,] = byhandadd

    # Interactive case: fixed
  intbeta.hats[j,] = mint1$coef
  intzeta.hats[j,] = mint2$coef
  intby.hand[j,] = byhandint

    # Interactive case with correlation
  intBbeta.hats[j,] = mintB1$coef
  intBzeta.hats[j,] = mintB2$coef
  intBby.hand[j,] = byhandintB
 }
# End of loop---------------------------------------------------------
# Saving output-----------------------------------------------------


# For the example of an additive pattern
dffadd<-data.frame(cbind(addby.hand,addbeta.hats,addzeta.hats))
colnames(dffadd)<-c("hf1","hf2","hg","uc","uf1","uf2","ug","cc","cf1","cf2","cg")
# For the example of an interactive pattern
dffint<-data.frame(cbind(intby.hand,intbeta.hats,intzeta.hats))
colnames(dffint)<-c("hf1","hf2","hg","uc","uf1","uf2","ug","cc","cf1","cf2","cg")
# For the example of an interactive pattern with correlation
dffintB<-data.frame(cbind(intBby.hand,intBbeta.hats,intBzeta.hats))
colnames(dffintB)<-c("hf1","hf2","hg","uc","uf1","uf2","ug","cc","cf1","cf2","cg")
# For p-values
dffpvalues<-data.frame(cbind(p1add,p2add,p1int,p2int,p1intB,p2intB))
colnames(dffpvalues)<-c("p1add","p2add","p1int","p2int","p1intB","p2intB")

# Figures ###################################################################
# Figures and correlations for additive patterns of the modelled data

# P values
dfpvalres<-data.frame(c("Additive","Additive","Interactive fixed","Interactive fixe
d","Interactive correlated","Interactive correlated"),
                      c("f1","f2","f1","f2","f1","f2"),rep(NA,6))
colnames(dfpvalres)<-c("model","fi vs g", "Percentage")
for (q in 1:6){
propt<-sum(ifelse(dffpvalues[,q]<0.05,1,0))*100/N
dfpvalres[q,3]<-propt
}
# Percentage of significant p-values (at alpha = 0.05)
grid.table(dfpvalres)
```

	model	fi vs g	Percentage
1	Additive	f1	5.4
2	Additive	f2	5.4
3	Interactive fixed	f1	100.0
4	Interactive fixed	f2	100.0
5	Interactive correlated	f1	98.7
6	Interactive correlated	f2	98.7

```
# This loop produces 3 groups of graphs:

# In a real case scenario there will be only one group.
boxf1add<-boxf2add<-boxf1int<-boxf2int<-boxf1intB<-boxf2intB<-list()
#  Start of loop --------

for (q in 1:3){
  q2=1+4*(q-1)
 cc1<-cor.test(dffadd[,q2],dffadd[,q2+2])
 cc2<-cor.test(dffadd[,q2+1],dffadd[,q2+2])
 object1<-paste(ifelse(q==1,"byhand",ifelse(q==2,"X-matrix","Z-matrix")),"r=",forma
t(cc1$estimate, digits=2))
 object2<-paste(ifelse(q==1,"byhand",ifelse(q==2,"X-matrix","Z-matrix")),"r=",forma
t(cc2$estimate, digits=2))
 mycol<-ifelse(q==1, "light blue", ifelse(q==2, "light green", "orange"))
 g1<-xyplot(dffadd[,q2+2]~dffadd[,q2]|object1,cex=1,pch=21, col="black",fill=mycol,
strip=strip.custom(bg="aliceblue"), ylab="g", xlab="f1")
 g2<-xyplot(dffadd[,q2+2]~dffadd[,q2+1]|object2, cex=1,pch=21, col="black",fill=myc
ol, strip=strip.custom(bg="aliceblue"),ylab="g", xlab="f2")

 cc3<-cor.test(dffint[,q2],dffint[,q2+2])
 cc4<-cor.test(dffint[,q2+1],dffint[,q2+2])
 object3<-paste(ifelse(q==1,"byhand",ifelse(q==2,"X-matrix","Z-matrix")),"r=",forma
t(cc3$estimate, digits=2))
 object4<-paste(ifelse(q==1,"byhand",ifelse(q==2,"X-matrix","Z-matrix")),"r=",forma
t(cc4$estimate, digits=2))
 g3<-xyplot(dffint[,q2+2]~dffint[,q2]|object3,cex=1,pch=21, col="black",fill=mycol,
strip=strip.custom(bg="aliceblue"),ylab="g", xlab="f1")
 g4<-xyplot(dffint[,q2+2]~dffint[,q2+1]|object4,cex=1,pch=21, col="black",fill=myco
l, strip=strip.custom(bg="aliceblue"),ylab="g", xlab="f2")
```

```
 cc5<-cor.test(dffintB[,q2],dffintB[,q2+2])
 cc6<-cor.test(dffintB[,q2+1],dffintB[,q2+2])
 object5<-paste(ifelse(q==1,"byhand",ifelse(q==2,"X-matrix","Z-matrix")),"r=",
format(cc5$estimate, digits=2))

 object6<-paste(ifelse(q==1,"byhand",ifelse(q==2,"X-matrix","Z-matrix")),"r=",
format(cc6$estimate, digits=2))

 g5<-xyplot(dffintB[,q2+2]~dffintB[,q2]|object5, cex=1,pch=21, col="black",
fill=mycol, strip=strip.custom(bg="aliceblue"),ylab="g", xlab="f1")

 g6<-xyplot(dffintB[,q2+2]~dffintB[,q2+1]|object6, cex=1,pch=21, col="black",fill=m
ycol, strip=strip.custom(bg="aliceblue"),ylab="g", xlab="f2")

boxf1add[[q]]<-g1
boxf2add[[q]]<-g2
boxf1int[[q]]<-g3
boxf2int[[q]]<-g4
boxf1intB[[q]]<-g5
boxf2intB[[q]]<-g6
}
# End of loop ----------
# Plots ---------------------------------------------------------------------
# Supplementary Figure 3
bbadd<-c(boxf1add[[1]],boxf2add[[1]],
      boxf1add[[2]],boxf2add[[2]],
      boxf1add[[3]],boxf2add[[3]], layout=c(2,3))
update(bbadd, as.table=TRUE,x.same=FALSE, ylab="Interactive effects", xlab="f1 (lef
t panels)     f2 (right panels)" )



# Supplementary Figure 4

bbint<-c(boxf1int[[1]],boxf2int[[1]],
         boxf1int[[2]],boxf2int[[2]],
         boxf1int[[3]],boxf2int[[3]], layout=c(2,3))
update(bbint, as.table=TRUE,x.same=FALSE, ylab="Interactive effects", xlab="f1 (lef
t panels)     f2 (right panels)")

# Supplementary Figure 5

bbintB<-c(boxf1intB[[1]],boxf2intB[[1]],
         boxf1intB[[2]],boxf2intB[[2]],
         boxf1intB[[3]],boxf2intB[[3]], layout=c(2,3))
update(bbintB, as.table=TRUE,x.same=FALSE)

############### END OF CODE ########################################
```

Supplementary note 4: Experimental design

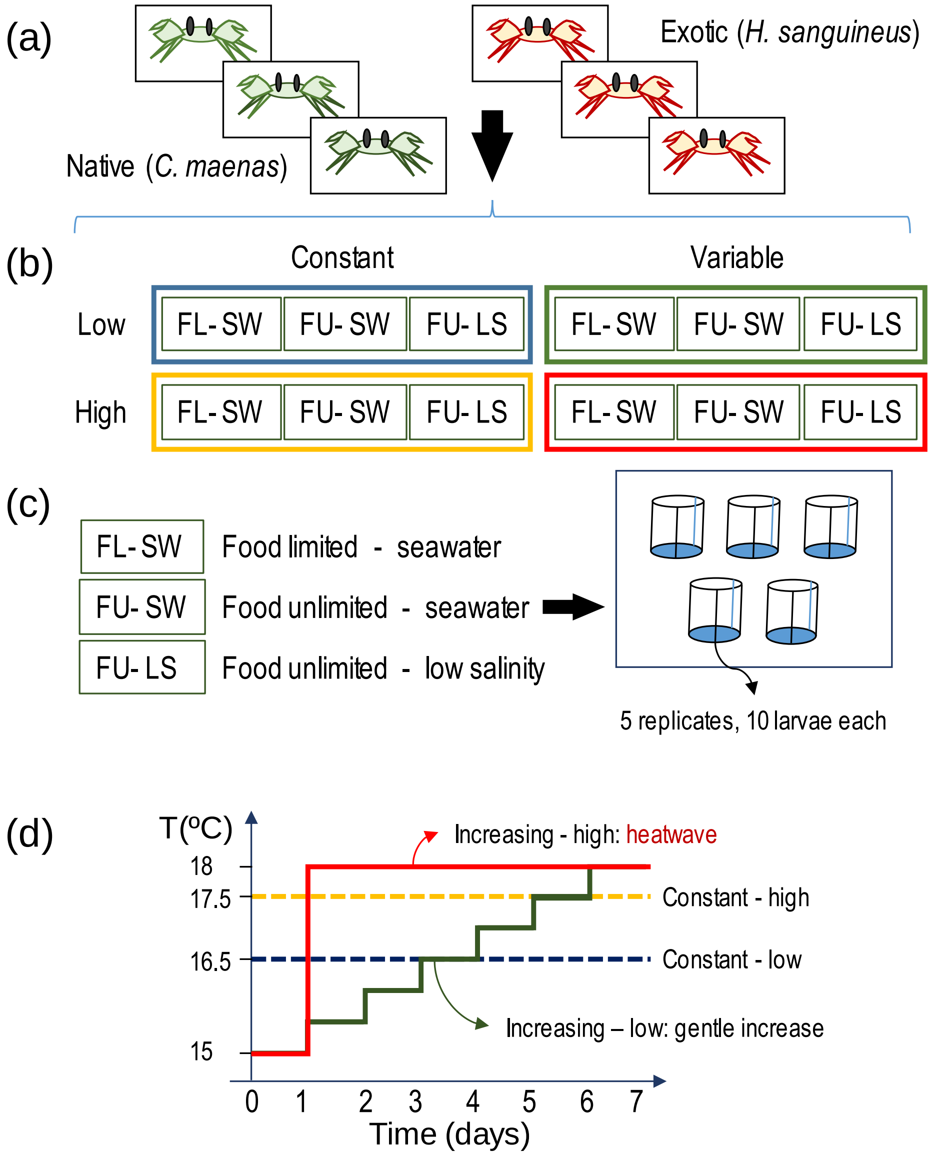

Supplementary Figure 6. Experimental design to study the effects of heatwaves on larval survival of a native (*Carcinus maenas*) and an exotic crab (*Hemigrapsus sanguineus*). (a) Larvae for experiments are obtained from 3 females from each species. (b) Larvae of each female are assigned to 4 temperature treatments: (1) Low and constant temperature; (2) Low and variable temperature (3) High and constant temperature, (4) Heatwave: High and variable temperature. (c) Larvae assigned to each group are then divided in subgroups to be exposed at three conditions of food and salinity. For each condition of food and salinity there are 5 replicate units of 10 larvae each. (d) Temperature profile for each of the four temperature treatments.

Supplementary Note 5: Experimental effects of heatwaves in an invasive and a native species

5.1. Tables

Supplementary Table 1. *Carcinus maenas*. Percent mortality after the increase in temperature experienced in day 1 in larvae reared under the simulated heatwave for larvae hatching from three different females (F1-F3).

	F1	F2	F3
Optimal food and salinity	36	55	100
Food limitation	82	72	67
Low salinity	100	59	100

Supplementary Table 2. *Carcinus maenas*: Summary of model selection (backward removal) evaluating the effects of food condition (F: fed 24 h *vs*. 6 h a day), temperature regime (R: constant or variable), temperature average (T: low= 16.5 °C, high = 17.5 °C), and female of origin (♀), on the proportion of larvae reaching the Zoea II. Model selection (based on AICc) occurred sequentially in order to determine the best variance and fixed structures. Models called "Reference" are intermediate candidate models containing only the higher interaction terms retained in the previous steps; they are used as reference for model selection of the subsequent lower level terms. AICc values in bold: terms retained through model selection; AICc values in red: indicate best models. The fixed structure of the best models is given at the bottom of the table. The symbol * means that terms combine in a factorial model (e.g. F*T=F+T+F:T).

Model selection: variance structure	AICc	
Term	log	logistic
R:T:F:♀	99	394
R	57	318
T	53	318
F	49	318
♀	**33**	316
H	53	**315**
Model selection: Fixed structure		
Full model: R*T*F*♀	-51	303
Terms removed		
R:T:F:♀	-57	299
R:T:F	**-49**	**304**
R:T:♀	-58	295
R:F:♀	-62	293
T:F:♀	-58	299
Reference-1	-64	290
F:♀	-62	285
T:♀	-66	286
R:♀	-68	285
Reference-2	-68	276
♀	**-9**	**342**
Best models (fixed structure)	AICc	
Log R*T*F + ♀	-68	
Logistic R*T*F + ♀	276	

Supplementary Table 3. *Hemigrapsus sanguineus*. Summary of model selection (backward removal) evaluating the effects of food condition (F: fed 24 h *vs*. 6 h a day), temperature regime (R: constant or variable), temperature average (T: low= 16.5 °C, high = 17.5 °C) and female of origin (♀), on the proportion of larvae reaching the Zoea II. Model selection (based on AICc) occurred sequentially in order to determine the best variance and fixed structures. Models called "Reference" are intermediate candidate models containing only the higher interaction terms retained in the previous steps; they are used as reference for model selection of the subsequent lower level terms. AICc values in bold: terms retained through model selection; AICc values in red: indicate best models. The fixed structure of the best models is given at the bottom of the table. The symbol * means that terms combine in a factorial model (e.g. F*T=F+T+F:T).

Model selection: variance structure	AICc	
Term	log	logistic
R:T:F:♀	6	378
R	-80	284
T	-80	284
F	-79	284
♀	-76	288
H	**-83**	**281**
Model selection: Fixed structure		
Full model: R*T*F*♀	-192	269
Terms removed		
R:T:F:♀	-198	264
R:T:F	-201	260
R:T:♀	-203	258
R:F:♀	-202	259
T:F:♀	**-191**	**268**
Reference-1	-210	251
R:♀	-215	247
R:F	-212	249
R:T	-212	250
Reference-2	-219	243
R	**-221**	**241**
Best models (fixed structure)	AICc	
Log R*F*♀	-221	
Logistic R*F*♀	241	

Supplementary Table 4. *Carcinus maenas*: Summary of model selection (backward removal) evaluating the effects of salinity (S: 20 or 32.5 PSU), temperature regime (R: constant or variable), temperature average (T: low= 16.5 °C, high = 17.5 °C) and female of origin (♀), on the proportion of larvae reaching the Zoea II. Model selection (based on AICc) occurred in two steps in order to determine the best variance structure and subsequently the best fixed structure. Terms retained after likelihood ratio test are as follows [1]LR=7.38, p=0.025; [2]LR=7.25, p=0.026; [3]LR=6.29, p=0.043. Other symbols as in Supplementary Table 2.

Model selection: variance structure	AICc	
Term	log	logistic
R:T:F:♀	87	407
R	22	330
T	23	331
S	23	331
♀	**18**	330
H	21	**328**
**Model selection: Fixed structure**		
Full model: R*T*S*♀	-70	318
Terms removed		
R:T:S:♀	-70	318
R:T:S	-72	315
R:T:♀	-74	314
R:S:♀	-73	313
T:S:♀	**-69**[1]	316
Reference-1	-79	306
S:♀	NA	**308**[2]
T:♀	NA	**307**[3]
R:♀	NA	302
R:S	-81	305
R:T	-81	304
T:S	-83	305
Reference-2	-87	297
R	**-90**	**296**
**Best models (fixed structure)**	AICc	
Log T*S*♀	-90	
Logistic T+S+♀+S:♀+ T:♀	296	

Supplementary Table 5. *Hemigrapsus sanguineus*: Summary of model selection (backward removal) evaluating the effects of salinity (S: 20 or 32.5 PSU), temperature regime (R: constant or variable), temperature average (T: low= 16.5 °C, high = 17.5 °C) and female of origin (♀), on the proportion of larvae reaching the Zoea II. Model selection (based on AICc) occurred in two steps in order to determine the best variance structure and subsequently the best fixed structure. Other symbols as in Supplementary Table 2. The best model was the null model.

Model selection: variance structure	AICc	
Term	log	logistic
R:T:F:♀	6	378
R	-119	299
T	-116	301
S	-116	300
♀	-114	304
H	-120	297
Model selection: Fixed structure		
Full model: R*T*S*♀	-240	281
Terms removed		
R:T:S:♀	-242	279
R:T:S	-244	276
R:T:♀	-247	274
R:S:♀	-246	274
T:S:♀	-244	276
Reference-1	-255	265
R:S	-255	265
R:T	-255	266
R:♀	-258	262
S:♀	-256	263
T:♀	-255	263
T:S	-258	262
Reference-2	-261	259
R	-263	257
	-263	257
T	-263	256
S	-262	258
Null	**-267**	**253**

5.2. Proportion survival discriminated by female of origin

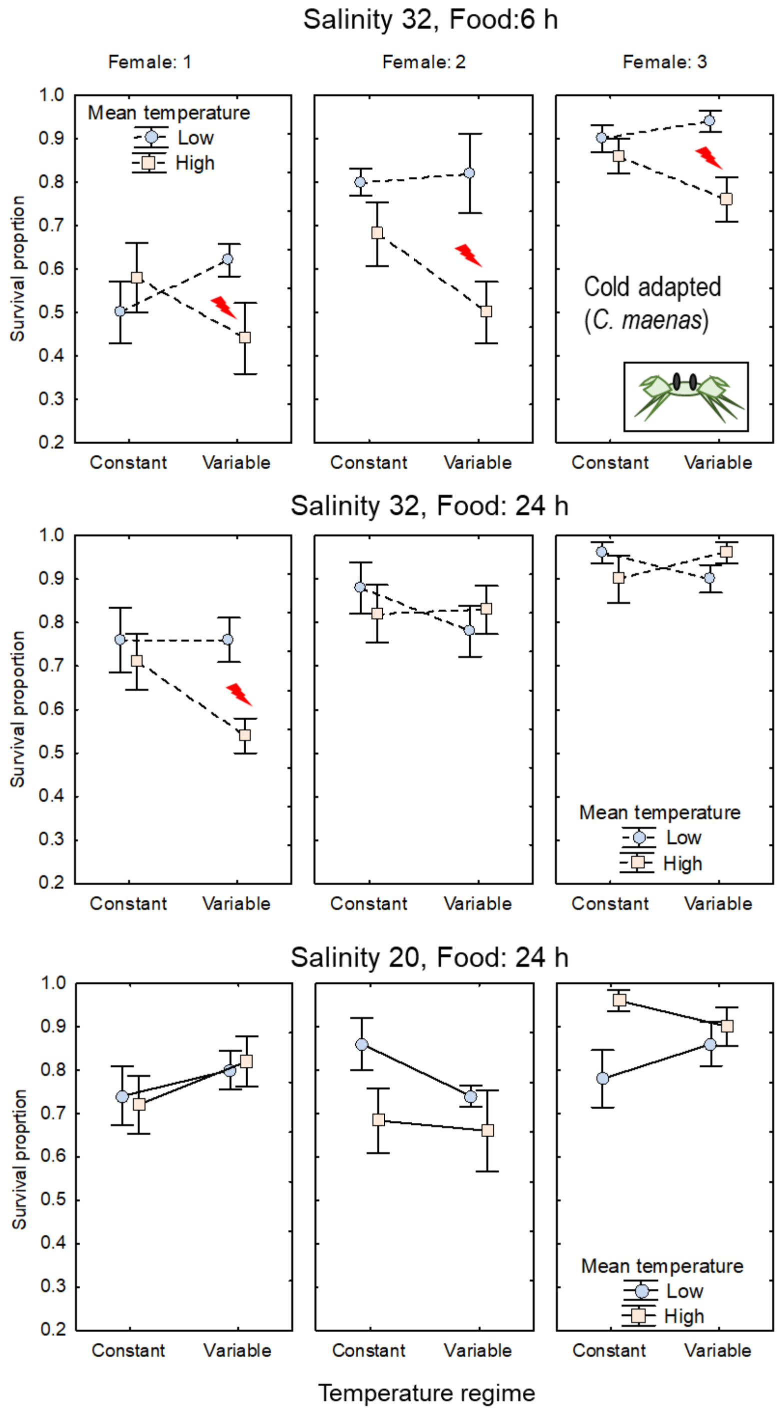

Supplementary Figure 7. Heatwave effect on survival of larvae of the crab *Carcinus maenas*, discriminated by female of origin. The overall significant effect of the heatwave treatment is indicated with a red arrow: it is different from a treatment with same average but constant temperature, and from a treatment with variable temperature but gentle variation. Bars are standard errors calculated from 5 replicate units.

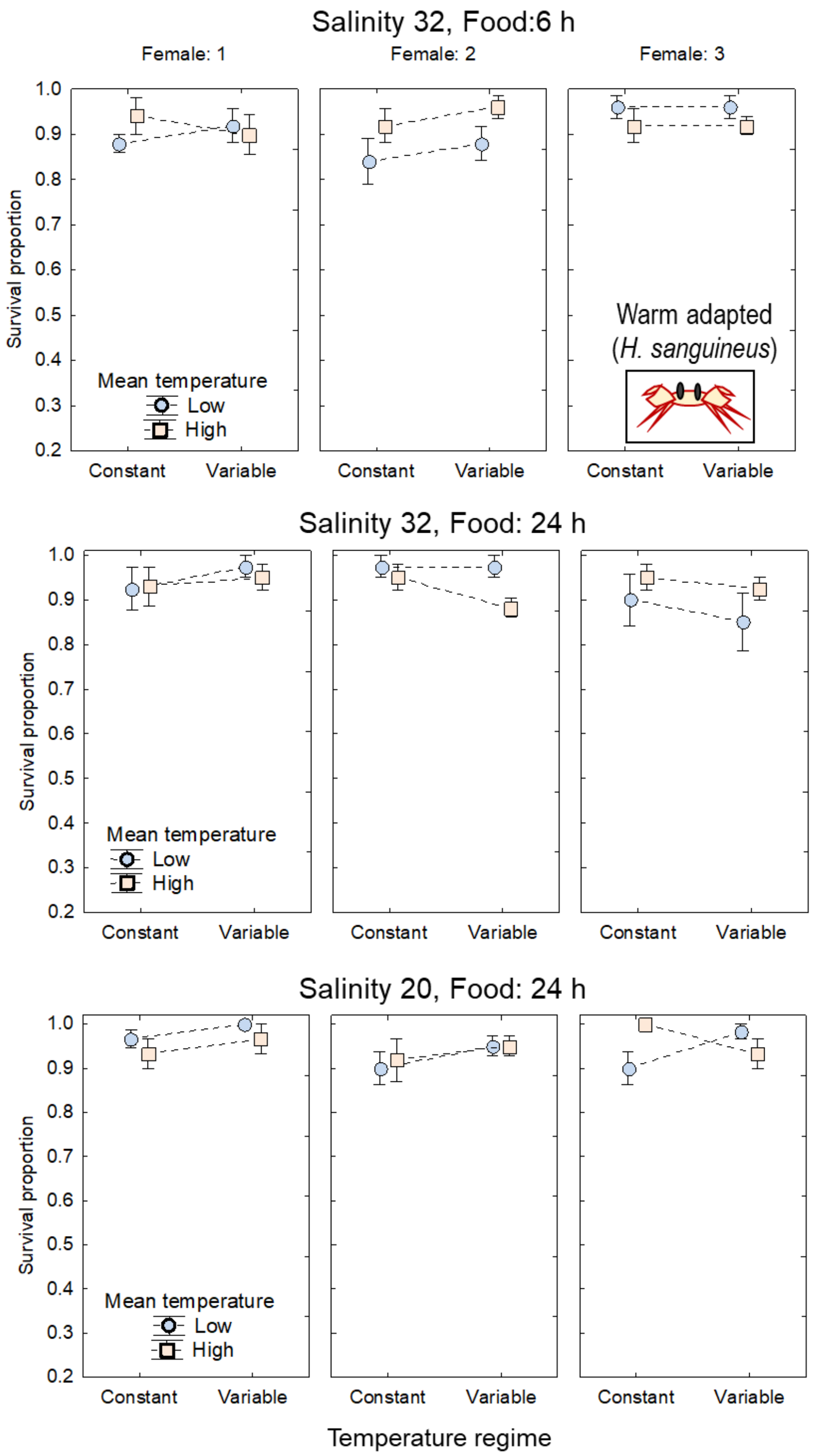

Supplementary Figure 8. Heatwave effect on survival of larvae of the crab *Hemigrapsus sanguineus*, discriminated by female of origin. The significant effect of the heatwave treatment is indicated with a red arrow: it is different from a treatment with same average but constant temperature, and from a

treatment with variable temperature but gentle variation. Bars are standard errors calculated from 5 replicate units.

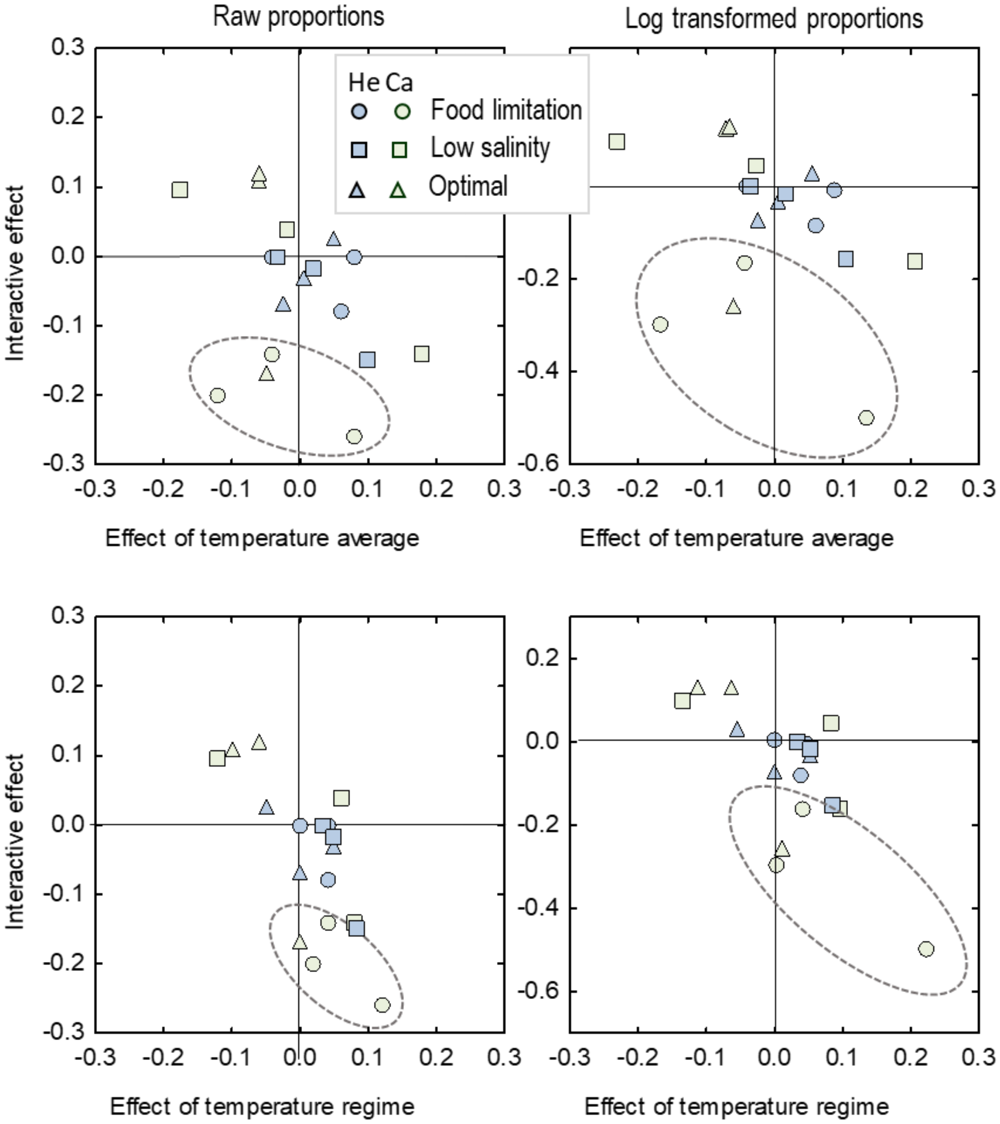

Supplementary Figure 9. Space-state representation of the survival responses in larvae exposed to the "heatwave treatment" for the crab *Carcinus maenas* (Ca, light green symbols) and the crab *Hemigrapsus sanguineus* (He, blue symbols). Left panels: representation based on raw proportions; right panels: log-transformed proportions (as in Fig. 6 of main text). Notice that the 3D representation is decomposed in 2D plots (upper and lower panels): x-axes give the contribution of temperature level (upper panels) or temperature variability (lower panels); the y-axes give the contribution of the interactive "heatwave effect" (i.e. an event characterised by high temperature in terms of average and variability. The reference condition was the treatment of low and constant temperature. Calculations were performed separately for the three environmental conditions: food limitation (but optimal salinity), optimal salinity and permanent access to food, low salinity (but permanent access to food). Each point represents an average response exhibited by a group of larvae produced by a specific female and exposed to a specific combination of food and salinity. Negative values in the interacting

effect correspond to decreases in survival under the heatwave treatment as compared to other temperature treatments. Dashed circles highlight responses to the simulated heatwave for *C. maenas*.

5.3. Alternative state-space representations

This section and Supplementary Figure 10 gives examples of alternative 2D-representations of the effect of the simulated heatwave on larval survival of *C. maenas* and *H. sanguineus* with focus on the effects of food limitation. For all cases, the calculations are based on a control treatment set by two factor combinations. Panels in **(a)** correspond to the representation in Fig. 6 of the main text. The reference condition was the combination corresponding to low and constant temperature (referred to as T_1 in the Methods section); there was one such reference for each food condition. Each point corresponds to larvae hatching from a particular female. Notice that the response under food limitation show the lowest values in the interactive axis reflecting the consistent negative effect of high average temperature and steep temperature increase. Also notice that there was also a case where such negative response was found for larvae (from female 1, see Supplementary Fig. 7) reared under *ad libitum* food conditions.

Panels in **(b)** use the treatment of low temperature and *ad-libitum* food as the reference; there was therefore separate references for the treatments of constant and increasing temperature. Panels in **(c)** use the treatment of temperature constant temperature and *ad libitum* food as the reference; there was therefore separate references for the treatments of low and high temperature. In both cases, scatterplots show that larvae of *H. sanguineus* are less responsive to those of *C. maenas* to the experimental conditions, as shown in **(a)**. In addition, one can see the strong negative effect of food limitation interacting with temperature average **(b)** or temperature regime **(c)** in survival of larvae of two females (F2 and F3): those effects corresponding to the heatwave conditions, as they are found when food limitation coincides with the scenario of high average temperature and steep temperature increase. Notice that for larvae hatching from one female (F1), the interactive effects are not strong but instead there is a main effect of high temperature or steep temperature increase: this reflects the fact that for F1, the heatwave effect was found both under food limitation and *ad libitum* food conditions (see Supplementary Fig. 7).

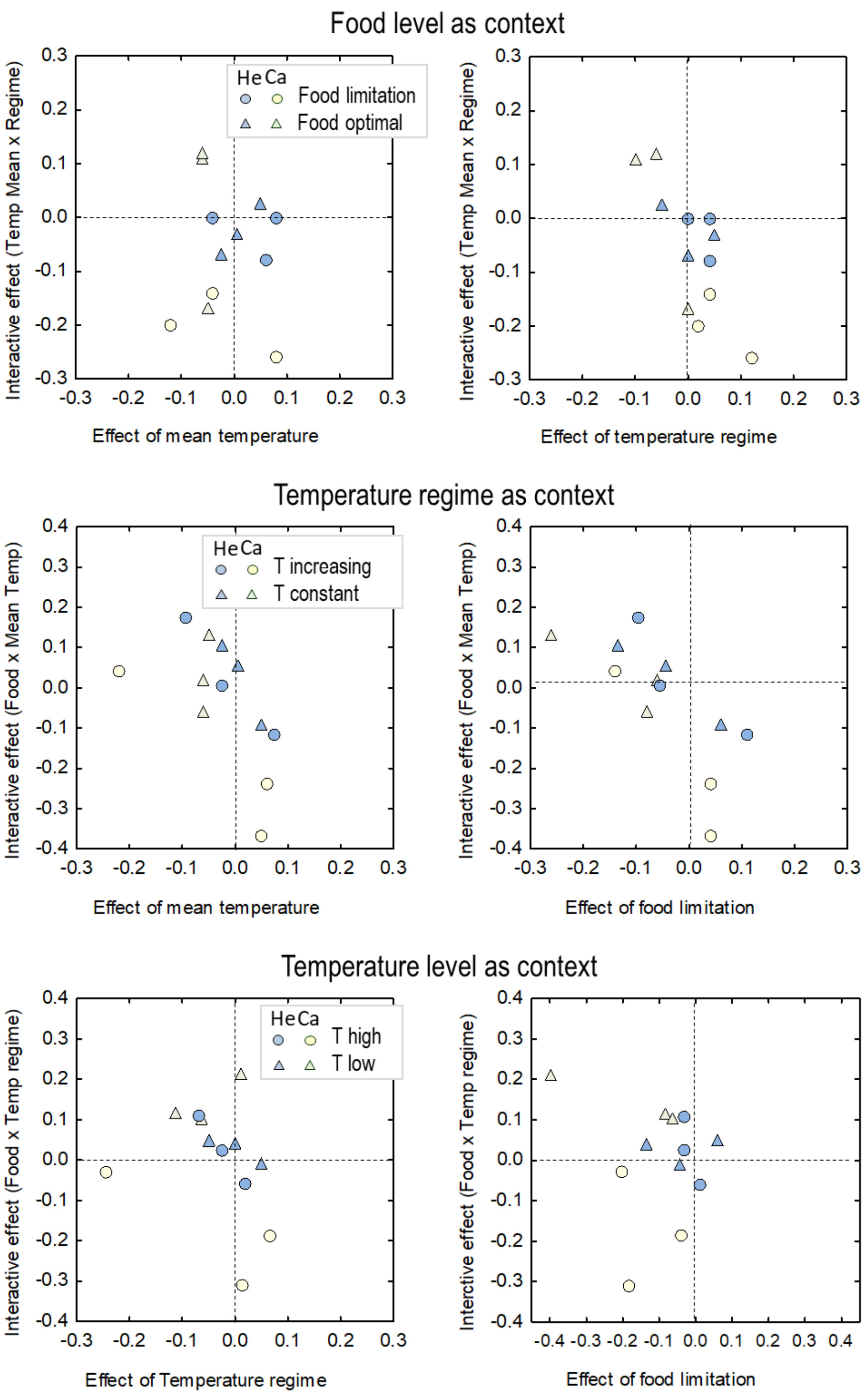

Supplementary Figure 10. Different space-state representations of the effect of heatwaves and food conditions on larval survival in the crab *Carcinus maenas* (Ca) and *Hemigrapsus sanguineus* (He). The top panels are as Fig. 6 of the main text (without the salinity treatment) showing the interacting effect of temperature regime and average in larvae reared at different food contexts. The mid panels show the interactive effect of temperature regime and food level in larvae reared under different conditions of temperature average. The bottom panels show the combined effect of temperature average and food level in larvae reared under different contexts of temperature regime.
