# Supplementary material for: A state-space approach to understand responses of organisms, populations and communities to multiple environmental drivers
Source: Commun Biol. 2021 Sep 30;4:1142. doi: 10.1038/s42003-021-02585-1 (PMC8484576; doi:10.1038/s42003-021-02585-1)
Supplement: Supplementary file 2 — Description of Supplementary Files [file 42003_2021_2585_MOESM2_ESM.pdf]

Description of Additional Supplementary Files

File name: Supplementary Data 1
Description: Data corresponding to figure 3 in manuscript
