# Supplementary material for: A state-space approach to understand responses of organisms, populations and communities to multiple environmental drivers
Source: Commun Biol. 2021 Sep 30;4:1142. doi: 10.1038/s42003-021-02585-1 (PMC8484576; doi:10.1038/s42003-021-02585-1)
Supplement: Supplementary file 4 — Reporting Summary [file 42003_2021_2585_MOESM4_ESM.pdf]

nature research

Corresponding author(s): BIO-21-0148-T

Last updated by author(s): Jul 21, 2021

Reporting Summary

Nature Research wishes to improve the reproducibility of the work that we publish. This form provides structure for consistency and transparency in reporting. For further information on Nature Research policies, see our Editorial Policies and the Editorial Policy Checklist.

Statistics

For all statistical analyses, confirm that the following items are present in the figure legend, table legend, main text, or Methods section.

n/a	Confirmed	
☐	☒	The exact sample size (n) for each experimental group/condition, given as a discrete number and unit of measurement
☒	☐	A statement on whether measurements were taken from distinct samples or whether the same sample was measured repeatedly
☐	☒	The statistical test(s) used AND whether they are one- or two-sided<br>*Only common tests should be described solely by name; describe more complex techniques in the Methods section.*
☐	☒	A description of all covariates tested
☐	☒	A description of any assumptions or corrections, such as tests of normality and adjustment for multiple comparisons
☐	☒	A full description of the statistical parameters including central tendency (e.g. means) or other basic estimates (e.g. regression coefficient) AND variation (e.g. standard deviation) or associated estimates of uncertainty (e.g. confidence intervals)
☐	☒	For null hypothesis testing, the test statistic (e.g. F, t, r) with confidence intervals, effect sizes, degrees of freedom and P value noted<br>*Give P values as exact values whenever suitable.*
☒	☐	For Bayesian analysis, information on the choice of priors and Markov chain Monte Carlo settings
☐	☒	For hierarchical and complex designs, identification of the appropriate level for tests and full reporting of outcomes
☒	☐	Estimates of effect sizes (e.g. Cohen's d, Pearson's r), indicating how they were calculated

Our web collection on statistics for biologists contains articles on many of the points above.

Software and code

Policy information about availability of computer code

Data collection	ata was collected in experiments and from the literature as specified in the section of methods and supp
Data analysis	Data was analysed using R and Rstudio using methods that are published in the literature (see methods). Separate R code was needed for simulations: that code is provided in the supplement

For manuscripts utilizing custom algorithms or software that are central to the research but not yet described in published literature, software must be made available to editors and reviewers. We strongly encourage code deposition in a community repository (e.g. GitHub). See the Nature Research guidelines for submitting code & software for further information.

Data

Policy information about availability of data

All manuscripts must include a data availability statement. This statement should provide the following information, where applicable:

- Accession codes, unique identifiers, or web links for publicly available datasets
- A list of figures that have associated raw data
- A description of any restrictions on data availability

ata from the experiment with Hemigrapsus larvae are available from the corresponding author on reasonable request; such data is stored in the database PANGEA . We are awaiting for the accession code. Additional data were taken from the literature as specified in the methods and supplement Section 2 and such data is provided as an Excel file in association with code used to produce part of the results

Field-specific reporting

Please select the one below that is the best fit for your research. If you are not sure, read the appropriate sections before making your selection.

☐ Life sciences ☐ Behavioural & social sciences ☒ Ecological, evolutionary & environmental sciences

For a reference copy of the document with all sections, see nature.com/documents/nr-reporting-summary-flat.pdf

Ecological, evolutionary & environmental sciences study design

All studies must disclose on these points even when the disclosure is negative.

Study description	Laboratory experiment quantifying the effect of temperatre food limitation and salinity on surival of larvae of crabs Carcinus maenas and Hemigrapsus sanguineus (Crustacea Decapoda Brachyura)
Research sample	A group of 10 larvae of the same origin (female and species) kept at a particular combination of temperature, salinity and access to food
Sampling strategy	There were 5 experimental units per treatment combination: this is the optimal number of replicates to be handled every day given the size of the experiment. We have achieved a larger number of replications by repeating the each experiment 3 times with larvae hatched from different females.
Data collection	Data was collected through an experiment which was carried out by all co-authors
Timing and spatial scale	NA as this is a a laboratory experiment
Data exclusions	No data were excluded from the analyses
Reproducibility	We repeated the experiment 3 times and all data of those repetitions were used and given in detail especially in the Supplement
Randomization	arvae from each female are assigned at random to treatments by mixing larvae in a glass bowl and pipetting them tot he different vials; vials are asigned subsequently
Blinding	Because we have to assign larvae to different salinites, temperatures and food conditions we cannot be blind to those conditions. Otherwise the experiment cannot be carried out.

Did the study involve field work? ☐ Yes ☒ No

Reporting for specific materials, systems and methods

We require information from authors about some types of materials, experimental systems and methods used in many studies. Here, indicate whether each material, system or method listed is relevant to your study. If you are not sure if a list item applies to your research, read the appropriate section before selecting a response.

Materials & experimental systems

n/a	Involved in the study	
☒	☐	Antibodies
☒	☐	Eukaryotic cell lines
☒	☐	Palaeontology and archaeology
☐	☒	Animals and other organisms
☒	☐	Human research participants
☒	☐	Clinical data
☒	☐	Dual use research of concern

Methods

n/a	Involved in the study	
☒	☐	ChIP-seq
☒	☐	Flow cytometry
☒	☐	MRI-based neuroimaging

Animals and other organisms

Policy information about studies involving animals; ARRIVE guidelines recommended for reporting animal research

Laboratory animals	N/A
Wild animals	We collected adult females of crabs Carcinus maenas and Hemigrapsus sanguienus in the intertidal of the island of Helgoland (North Sea, Germany). Females were kept in aquaria under optimal conditions of temperature, salinity and food and they did not participate in the experiment. Experiments were carried out with the larvae produced by those females.
Field-collected samples	N/A

Ethics oversight	The research presented in this paper complies with the guidelines from the directives 2010/63/EU of the European parliament and of the Council of 22nd September 2010 on the protection of animals used for scientific purposes. All applicable international, national, and/or institutional guidelines for the care and use of animals were followed.

Note that full information on the approval of the study protocol must also be provided in the manuscript.
